# Focal hepatic lesions in Gd-EOB-DTPA enhanced MRI: the atlas

**DOI:** 10.1007/s13244-012-0179-7

**Published:** 2012-06-15

**Authors:** José Traila Campos, Claude B. Sirlin, Jin-Young Choi

**Affiliations:** 1 Department of Radiology, FMUP, Hospital S. João, Alameda Prof. Hernâni Monteiro, 4200-319 Porto, Portugal; 2 Department of Radiology, UCSD, San Diego, USA; 3 Department of Radiology, Yonsei University Health System, Seoul, Republic of Korea

**Keywords:** Magnetic resonance, MR, Gd-EOB-DTPA, Liver

## Abstract

**Objective:**

This article reviews the different technical aspects and pitfalls of gadolinium (Gd)-ethoxibenzyl (EOB)-diethylenetriamine pentaacetic acid (DTPA) and the advantages of the hepatocellular phase (HCP) and defines its specific imaging features of liver lesions.

**Background:**

Gd-EOB-DTPA is a contrast agent with combined properties of a conventional non-specific extracellular and a hepatocyte-specific contrast agent. Benign cirrhosis-associated nodules are characterised by isointensity in dynamic imaging and the HCP. Hepatocellular carcinomas (HCCs) usually show hyperenhancement in the arterial phase, with washout in the portal vein pressure (PVP) and hypointensity in the HCP. Among other characteristic findings, we have the mosaic pattern, a capsule, the “nodule-in-nodule” appearance and the satellite nodules. The fibrolamellar HCC is a large enhancing heterogeneous lesion, on a non-cirrhotic liver, with a hypointense scar in every sequence. THIDs (transient hepatic intensity differences) are perfusional alterations, characterised by hyperintensity in the arterial phase, with no alterations in the rest of the sequences including the HCP. Adenoma and focal nodular hyperplasia (FNH) are lesions, occurring more frequently in young women, with brisk arterial enhancement, differentiated by the scar and the uptake of Gd-EOB-DTPA in the HCP. Focal eosinophilic infiltration is a difficult diagnosis, with characteristics such as a non-spherical shape and irregular borders suggesting it. Besides these lesions, in which Gd-EOB-DTPA has a clear advantage, there are a few where the specificities of this agent can be troublesome: haemangiomas, focal fat/sparing, foreign body reaction, cholangiocarcinoma and metastases.

**Conclusion:**

Gd-EOB-DTPA is comparable to extracellular agents, and uptake by functioning hepatocytes in the delayed phase provides additional information that further improves detection and characterisation of many hepatic lesions.

***Main Messages*:**

- *Gd-EOB-DTPA offers advantages for the imaging of many liver lesions including HCC, fibrolamellar HCC, FNH and adenoma.*
- *The properties of Gd-EOB-DTPA can pose problems when dealing with haemangiomas, cholangiocarcinoma and metastases among others.*
- *The uptake of Gd-EOB-DTPA by functioning hepatocytes in the delayed phase provides additional information that further improves detection and characterisation of many hepatic lesions.*

## Introduction

Hepatocyte-specific magnetic resonance (MR) imaging contrast agents were developed for detection and characterisation of focal liver lesions. Currently, there are two hepatocyte-specific agents available: gadolinium (Gd)-ethoxibenzyl (EOB)-diethylenetriamine pentaacetic acid (DTPA) and gadobenate dimeglumine (Gd-BOPTA). Gd-EOB-DTPA has greater hepatocellular uptake and biliary excretion than Gd-BOTPA and is the focus of this review.

**Fig. 1:**
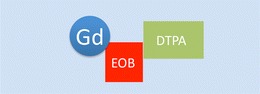
Molecular structure of Gd-EOB-DTPA

Gd-EOB-DTPA—known generically as gadoxetic acid and commercially as Eovist (United States) or Primovist (most parts of the World) (Bayer Healthcare)—is a highly water-soluble contrast agent in which a lipophilic EOB group is attached to gadolinium (Gd)-DTPA (Fig. 1). The resulting compound has properties of a conventional non-specific extracellular contrast agent in the vascular phases after administration, permitting assessment of tissue vascularity, and those of a hepatocyte-specific agent in the more delayed phases, allowing assessment of hepatocellular uptake and biliary excretion [1, 2]. Due to the presence of the lipophilic EOB group, Gd-EOB-DTPA is actively transported from the sinusoidal space into hepatocytes via organic anion transporting polypeptides (OATPs). Entry of the contrast agent into liver cells causes intense parenchymal enhancement, beginning within 1 or 2 min of contrast agent injection. The enhancement peaks at around 20 min and lasts for at least 2 h. The agent is excreted from hepatocytes into the biliary canaliculi via multidrug resistance-associated proteins (MRPs) [3, 4]. Excretion into the bile ducts causes biliary luminal enhancement, as early as 5-10 min after injection in normal individuals. It is eliminated in approximately equal proportions by the liver, via hepatocellular uptake with subsequent biliary excretion (43.1–53.2%), and by the kidneys (41.6–51.2%) [5, 6].

Gd-EOB-DTPA has been shown to be comparable to non-specific extracellular agents for lesion detection and characterisation in the vascular imaging phases. Moreover, uptake by functioning hepatocytes in the delayed phase provides additional information that further improves detection and characterisation of many hepatic lesions, reported in recent articles [7, 8]. The purpose of this review is to illustrate the typical appearance of focal hepatic lesions with MRI and to understand the advantages, pitfalls and differential diagnosis using Gd-EOB-DTPA. The lesions are thus divided into those for which Gd-EOB-DTPA demonstrates advantages, those in which have pitfalls and those in which the lesion behaviour is equivalent to that of the use of an extracellular fluid (ECF) contrast agent.

## General considerations

The vascular imaging phases using Gd-EOB-DTPA are similar to those using extracellular agents with a few caveats:

1. Arterial phase enhancement of vessels and lesions with Gd-EOB-DTPA tends to be less intense than with extracellular agents due to the following factors. Gd-EOB-DTPA is administered in a smaller dose than extracellular agents (0.025 mmol/kg), providing a shorter temporal window to acquire images during peak arterial enhancement. Additionally, there is a higher frequency of imaging artefacts in the arterial phase when compared with extracellular agents. The cause of these artefacts is not well understood. These “truncation” artefacts may occur due to abrupt concentration changes during high spatial k-space filling, and may alter the intensity, shape and anatomical detail of the structures.
2. Hepatocellular uptake begins almost immediately, leading to rapidly progressive liver enhancement. Consequently, there is no pure venous or equilibrium phase. Because the liver enhances sooner and more intensely than with extracellular agents, the intensity of the lesions relative to the liver may appear different. For example, blood vessels and blood-pool lesions, such as haemangiomas, which remain hyperintense in comparison to the liver during all vascular phases with extracellular agents, appear to “wash out” with Gd-EOB-DTPA and, after 5-10 min, become hypointense.
3. The degree of hepatocellular enhancement depends on liver function, which roughly correlates with serum bilirubin levels, which is not a perfect predictor. In livers with good hepatic function, intense enhancement occurs. In livers without good function, due to cholestasis or hepatocellular dysfunction, enhancement of liver parenchyma may be weak. In such cases, blood vessels and blood pool lesions become isointense rather than hypointense in comparison to the liver.

## Focal hepatic lesions

### Lesions in which Gd-EOB-DTPA provides advantages

#### Cirrhosis-related nodules

**Fig. 2:**
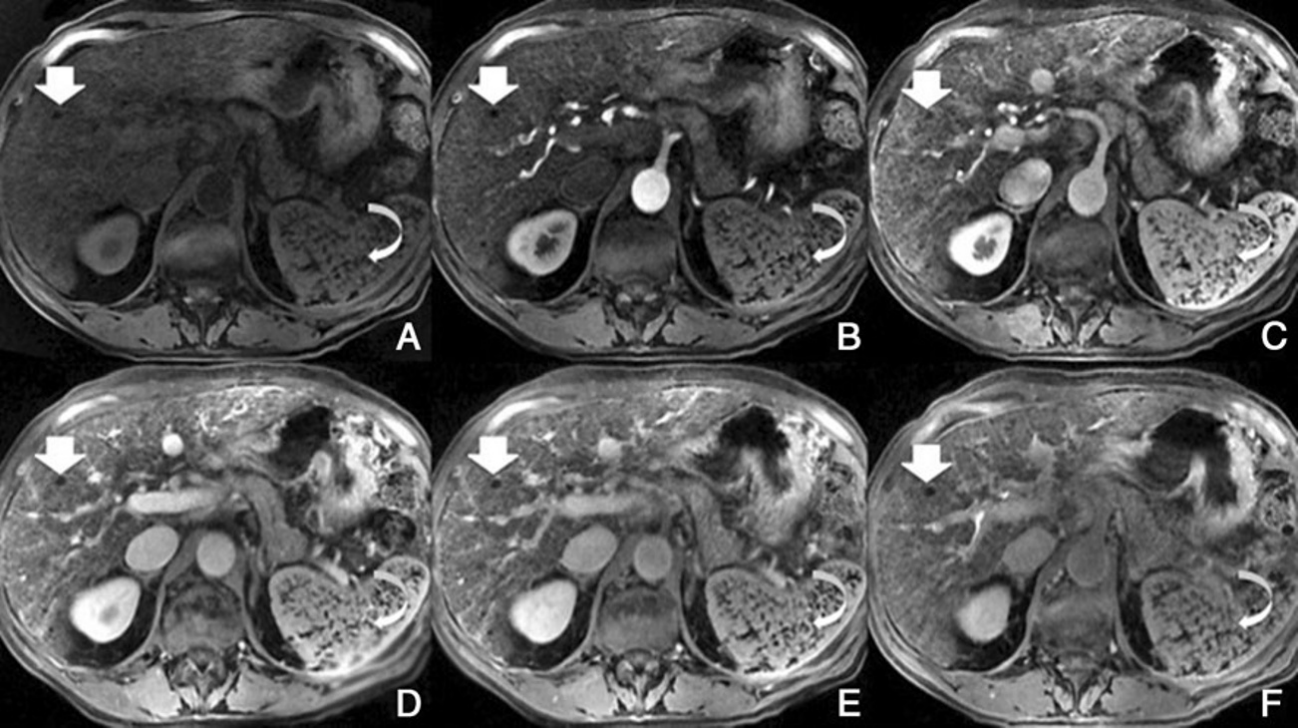
A benign cirrhosis-associated nodule in a 38-year-old woman with chronic liver disease. **a** Pre-contrast MRI, **b, c** arterial phase, **d** PV phase, **e** delayed phase, **f** hepatocellular phase. The image shows a liver with a lobulated contour and a nodular parenchyma (cirrhosis). The *solid arrows* point to a small rounded focal lesion, markedly hypointense in all sequences (more conspicuous with the increase of the TE), showing no enhancement with the contrast agent. The *curved arrow* points to multiple Gamna-gandy bodies (hemosiderin deposition)
  *Dynamic imaging with extracellular contrast agent*: After the injection of the contrast, most benign cirrhosis associated-nodules show similar enhancement to the adjacent liver in the arterial and venous phases [9, 10]. Some benign cirrhosis associated-nodules enhance in the arterial phase and typically fade to isointensity in the venous phases.
  *Imaging with Gd-EOB-DTPA*: Uptake and excretion of gadobenate dimeglumine by these nodules is usually preserved. Consequently, they are usually isointense in the hepatocellular phase (HCP) [11].
  *Differential diagnosis*: The diagnosis of most benign cirrhosis-associated nodules is straightforward with MRI, using extracellular agents. However, some benign cirrhosis associated-nodules may show enhancement in the arterial phase. These hypervascular benign nodules usually fade to isointensity in the venous phases. While most cirrhosis-associated nodules with arterial phase hyperenhancement and venous phase isoenhancement are benign, small hepatocellular carcinomas (HCCs) can have a similar appearance. Nodules with arterial phase hyperenhancement and venous phase isoenhancement can therefore cause diagnostic confusion and lead to inappropriate management. Use of Gd-EOB-DTPA can be helpful in the differential diagnosis of these lesions. Homogeneous isointensity in the HCP relative to liver suggests benignancy: routine follow-up is sufficient. By comparison, hypointensity in the HCP suggests malignancy: such lesions require biopsy, empirical treatment or close follow-up depending on lesion size and clinical considerations.
  *Potential pitfalls*: Siderotic and steatotic nodules may appear hypointense in the HCP, potentially mimicking early HCC. This is due to the iron and fat content being already of low signal intensity in the pre-contrast fat saturated sequences. Some benign nodules may appear hyperintense in the HCP [14] (Fig. 3). The mechanism is still unknown, but overexpression of organic anionic transporter peptides (OATP) or down-regulation of MDRs could possibly play a role. Such lesions may be mistaken for focal nodular hyperplasia (FNH)-like lesions (these are also benign, so the distinction is irrelevant) or atypical HCCs with HCP hyperintensity. The differentiation between benign and malignant HCP-hyperintense nodules is discussed in the following two sections. Small benign cirrhosis-associated nodules with arterial-phase hyperenhancement and HCP hypointensity may be indistinguishable from small HCCs.

1. Regenerative and dysplastic nodules
  *Overview*: Cirrhosis is pathologically defined by extensive fibrosis, nodular regeneration and architectural distortion of the parenchyma. The vast majority of cirrhosis-related nodules exhibit regenerative changes without cellular atypia and are known as regenerative nodules (RNs). A minority have dysplastic features and are known as dysplastic nodules (DNs). The differentiation between RN and DN is unreliable when attempted non-invasively, difficult with histopathology, and is of minor, if any, clinical relevance. For these reasons, these two entities are discussed together as benign cirrhosis-associated nodules.
  *Unenhanced MRI features*: Typically, benign cirrhosis-associated nodules are <2 cm and isointense to hyperintense on T1-weighted images, isointense to hypointense on T2-weighted images and isointense on diffusion-weighted (DW) images. Lipid-containing nodules or steatotic nodules display signal loss on out-of-phase gradient echo (GRE) images in comparison with in-phase images. Iron-containing nodules or siderotic nodules appear markedly hypointense on T2-weighted and T2*-weighted images (Fig. 2).

**Fig. 3:**
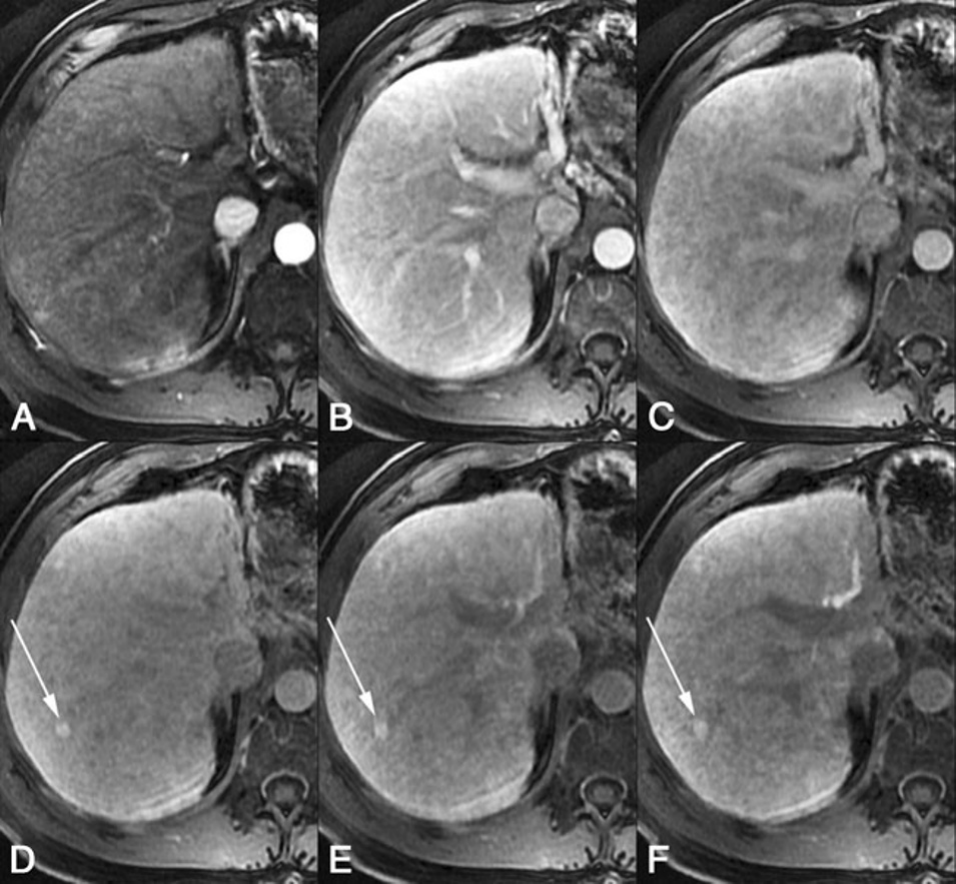
A benign cirrhosis-associated nodule in a 39-year-old man with chronic liver disease. Gd-EOB-DTPA enhanced MRI. **a** arterial phase, **b** PV phase, **c** delayed phase, **d** 5 min, **e** 10 min, **f** hepatocellular phase. The image shows a liver with a nodular contour and a heterogeneous parenchyma. The *arrows* point to a small rounded focal lesion accumulating Gd-EOB-DTPA- benign nodule
2. Acquired FNH-like lesions
  *Overview*: These lesions are described in cirrhotic livers, and are macroscopically, microscopically and immunohistochemically identical to classic FNH seen in non-cirrhotic livers. They are believed to originate from acquired hyperplastic responses to vascular alterations associated with cirrhosis, as opposed to conventional FNHs, which are believed to originate from developmental responses to congenital arterio-vascular malformations.
  *Unenhanced MRI features*: Hypointense to isointense on T1-weighted MR images and mildly hyperintense to isointense on T2-weighted images. The “central scar”, if present, is hyperintense on T2-weighted images.
  *Dynamic imaging with extracellular contrast agent*: These lesions usually show intense enhancement in the arterial phase and fade to isointensity in the portal venous and later phases. Rarely, they may reveal a slight washout in the PV phase; marked washout is incompatible with FNH. [12].
  *Imaging with Gd-EOB-DTPA*: Usually it enhances in the hepatobiliary phase (Fig. 4), but there have been reports of a few cases revealing hepatobiliary hypointensity [13].

**Fig. 4:**
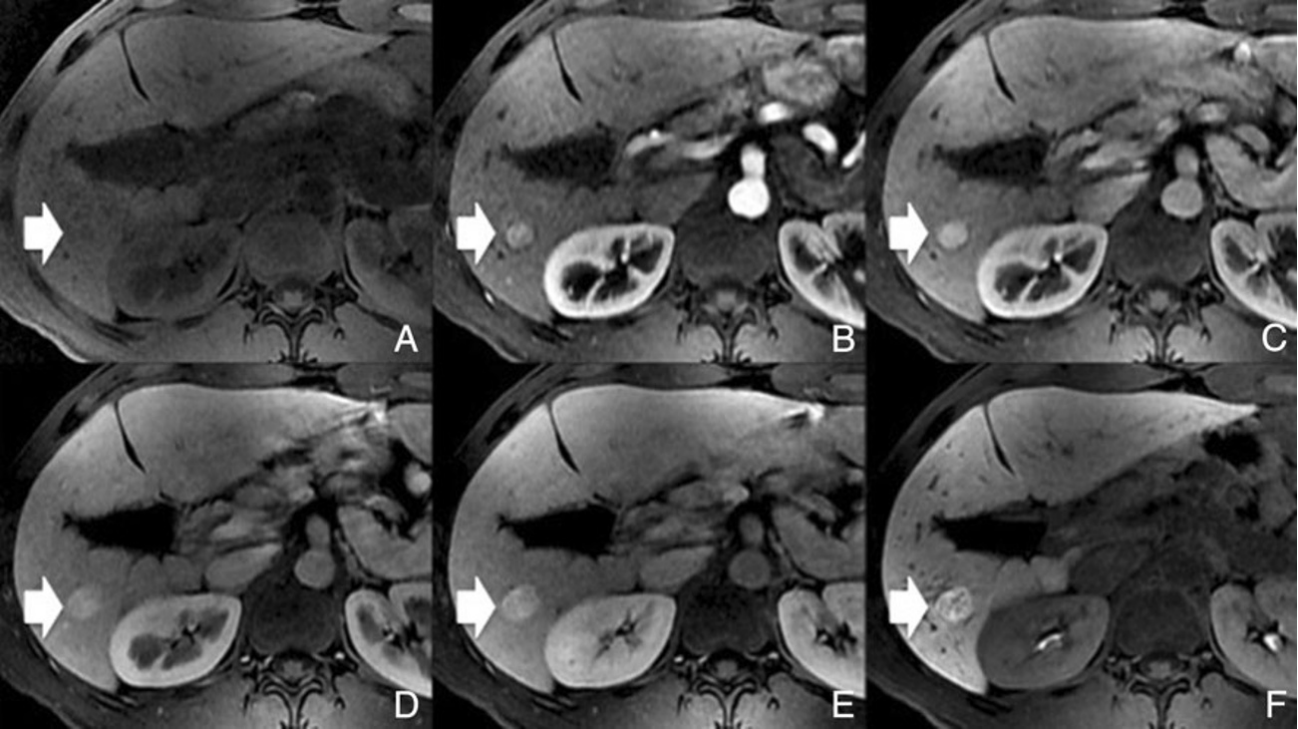
An FNH-like lesion in a 41-year-old man with chronic liver disease. **a** Pre-contrast MRI, **b** arterial phase, **c** PV phase, **d** 5 min, **e** 10 min, **f** hepatocellular phase. The image shows a liver with a lobulated contour. The *arrows* point to a small rounded focal lesion, isointense in the Pre-contrast sequence, showing brisk arterial enhancement. In the HCP it accumulates Gd-EOB-DTPA, exposing the typical FNH internal architecture
  *Differential diagnosis*: The major differential diagnosis is HCC, especially for small lesions with less than 3 cm, those without a central scar and those with HCP hypointensity.
  *Potential pitfalls*: While HCP hyperintensity is characteristic of FNH-like lesions, this finding is non-specific and may also be observed in HCCs (see below). Favouring the diagnosis of a FNH-like lesion is the presence of a central scar and/or a network of fibrous septa carving the nodule into smaller lobules. Favouring HCC are the mosaic architecture, presence of a tumour capsule, mildly hyperintense signal intensity of T2-weighted, restricted diffusion on DW images or a combination of these.
  While most FNH-like lesions exhibit diffuse HCP hyperintensity, these lesions may have central zones of portal venous phase washout and HCP hypointensity, mimicking HCC. In equivocal cases, close follow-up or biopsy should be considered (depending on lesion size and other factors).

#### Hepatocellular carcinoma

##### Overview

HCC is the most common primary malignant hepatic tumour. Underlying cirrhosis from viral hepatitis (B and C), alcoholism, non-alcoholic steatohepatitis (NASH) and toxin exposure are the predominant causal factors. Tumour markers, such as serum-fetoprotein or PIVKA-II, are usually elevated in patients with HCC [14, 15].

However, elevation of tumour markers does not help determine which of several lesions is malignant, if multiple nodules are observed, and negative tumour markers do not exclude HCC.

##### Unenhanced MRI features

**Fig. 5:**
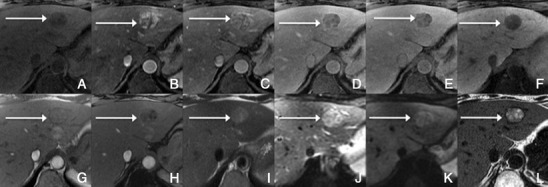
Fatty HCC in a 45-year-old man with chronic liver disease. **a** Pre-contrast MRI, **b, c** arterial phase, **d** 3 min, **f** 4 min, **f** 8 min, **g** IP, **h** OP, **i** SSFSE, **j** b0, **k** b500, **l** T1-weighted PDFF. The *arrows* point to a rounded focal lesion, with arterial enhancement, PV washout and hypointensity in the HCP. It also shows heterogeneous loss of signal in OP (fat), expressed likewise in the fat fraction map as bright areas in the lesion

On T1-weighted MR images, HCC is most often hypointense relative to the liver, although hyperintense lesions or areas of hyperintensity within hypointense lesions may be seen [15]. On T2-weighted images, HCC is generally hyperintense, although some well-differentiated HCC may be seen as isointense or mildly hypointense lesions. Steatotic HCCs are characterised by a signal loss on out-of-phase images relative to in-phase images (Fig. 5). Haemorrhagic hepatocellular carcinomas may have marked high signal intensity on T1-weighted images and low signal intensity on T2- and T2*-weighted images.

##### Dynamic imaging with extracellular contrast agent

**Fig. 6:**
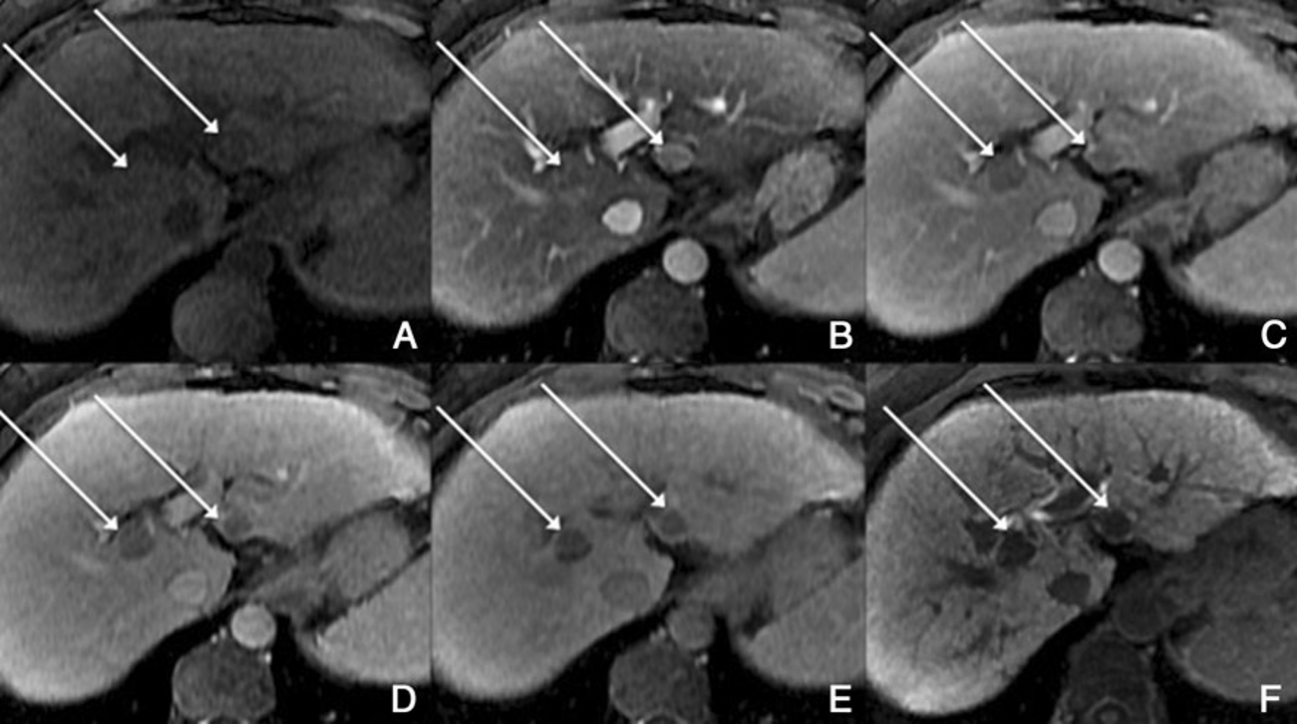
Hypervascular HCCs in a 50-year-old man with chronic liver disease. **a** Pre-contrast MRI, **b** arterial phase, **c** portal vein pressure (PVP), **d** 3 min, **f** 4 min, **f** HCP. Marked heterogeneous liver with nodular contour, with reticulation in the HCP- cirrhotic The *arrows* point to two rounded focal lesions, with arterial enhancement (one less prominent), PV washout and hypointensity in the HCP

**Fig. 7:**
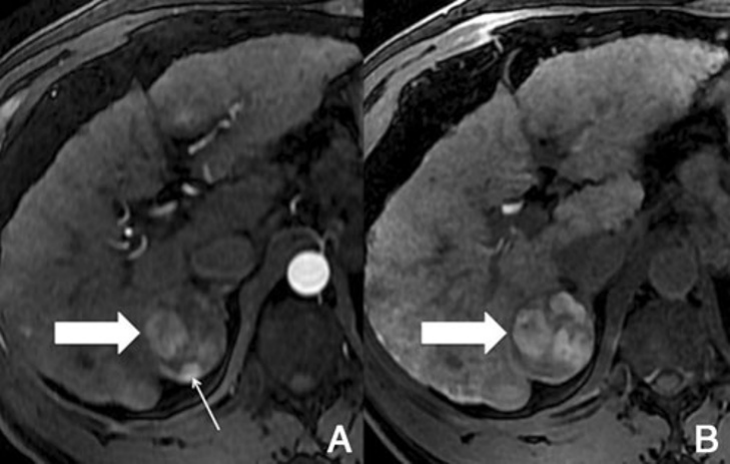
A 52-year-old man with chronic liver disease. **a** Arterial phase, **b** HCP. Imaging findings of a cirrhotic liver, with a lesion (*large arrows*) with areas of enhancement (*thin arrow*) and non-enhancing areas—mosaic pattern. In the HCP we found a similar appearance with areas taking up Gd-EOB-DTPA and others not

Typically, HCCs (80%) show intense enhancement in the arterial phase contrast-enhanced images, with washout in the portal venous phase (Fig. 6). Less than 20% of the HCCs are hypovascular (usually well-differentiated tumours) [15, 16]. Small HCCs (most commonly well differentiated HCCs) are usually homogeneous. Large HCCs (most commonly moderately or poorly differentiated) may exhibit a broad spectrum of morphologic features, including a mosaic pattern (Fig. 7), a tumour capsule, an intratumoural nodule (“nodule-in-nodule” appearance), and an extracapsular extension with the formation of satellite nodules. A tumour capsule shows higher enhancement than that of the surrounding parenchyma on delayed phase images. Capsular disruption, with extracapsular extension or absence of a capsule, is associated with poorer prognosis.

##### Imaging with Gd-EOB-DTPA

**Fig. 8:**
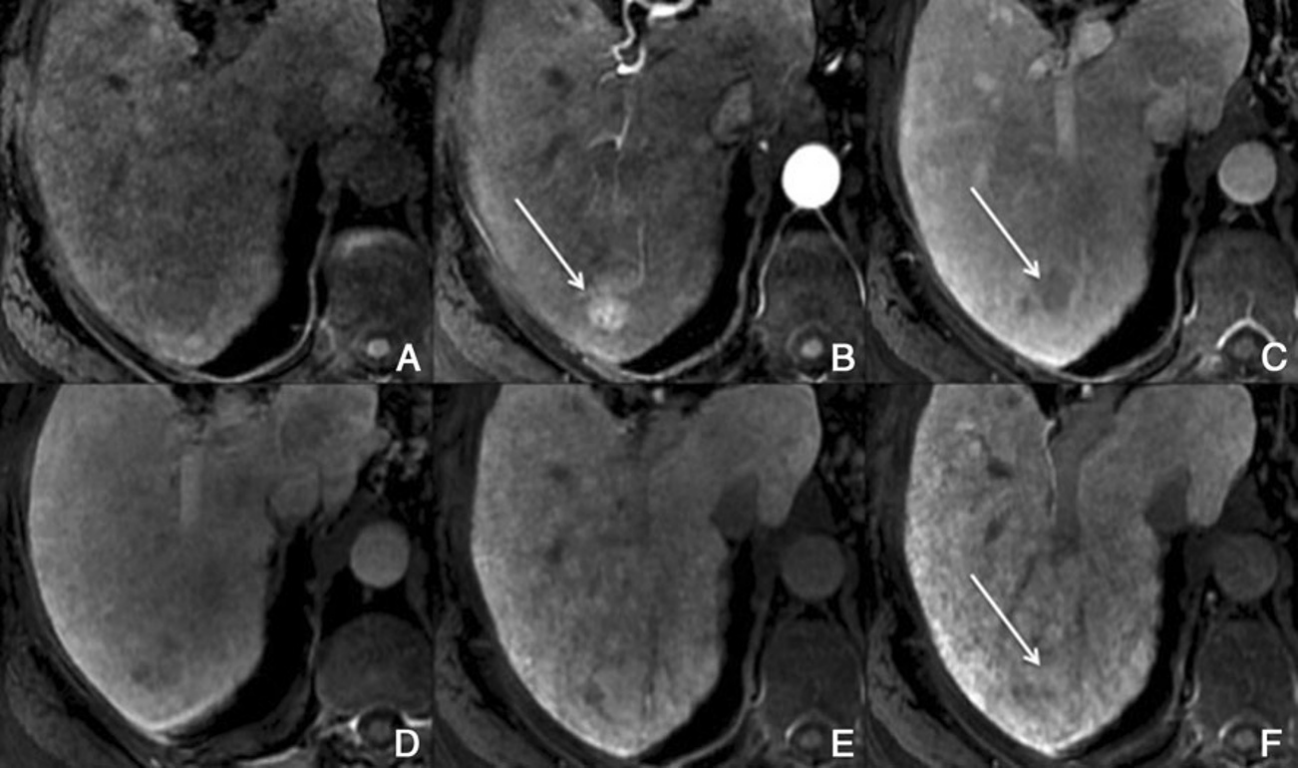
HCC in a 42-year-old woman with chronic liver disease. **a** Pre-contrast MRI, **b** arterial phase, **c** PV phase, **d** 5 min, **e** 10 min, **f** HCP. Hypervascular HCC, with washout in the PV phase and uptake of Gd-EOB-DTPA (*arrows*)

In the hepatocellular phase, HCCs usually show low signal intensity. The degree and homogeneity of hypointensity is variable, depending on the concentration and function of cellular membrane receptors and transporters (OATPs and multidrug resistance-associated proteins [MRPs]). In some (2.5-8.5%), uptake of the contrast agent can paradoxically occur (Fig. 8). Most of these hyperintense HCCs are well or moderately differentiated, with bile-producing or bile-containing capacity maintained [17–20].

##### Differential diagnosis

Cholangiocarcinoma, arterioportal (AP) shunt, atypical haemangioma and hepatic adenoma are possible diagnosis. The usual occurrence in a cirrhotic liver, in addition to the dynamic and hepatocelullar phase imaging, usually permit the differential diagnosis.

##### Potential pitfalls

Some HCCs will uptake Gd-EOB-DTPA in the hepatobiliary phase. These can usually be differentiated from RN, DN and FNH-like lesions because of their heterogeneity or mosaic pattern, the nodule-in-nodule appearance, the presence of a hypoenhancing rim (capsule) or the absence of a scar.

Infiltrative HCC may manifest as subtle heterogeneity and mild ill-defined hypointensity in the HCP. This may mimic background cirrhotic parenchyma and escape detection.

#### Fibrolamellar HCC

##### Overview

Fibrolamellar HCC is an uncommon type of HCC affecting young adults.

##### Unenhanced MRI features

It is usually seen as a large, well-circumscribed focal lesion with low signal intensity on T1-weighted MR images and high signal intensity on T2-weighted images. A central radiating scar is seen in 80% of cases and has low signal intensity on T1- and T2-weighted images. Areas of liquefactive necrosis may occur centrally and appear markedly hyperintense on T2-weighted images.

##### Dynamic imaging with extracellular contrast agent

**Fig. 9:**
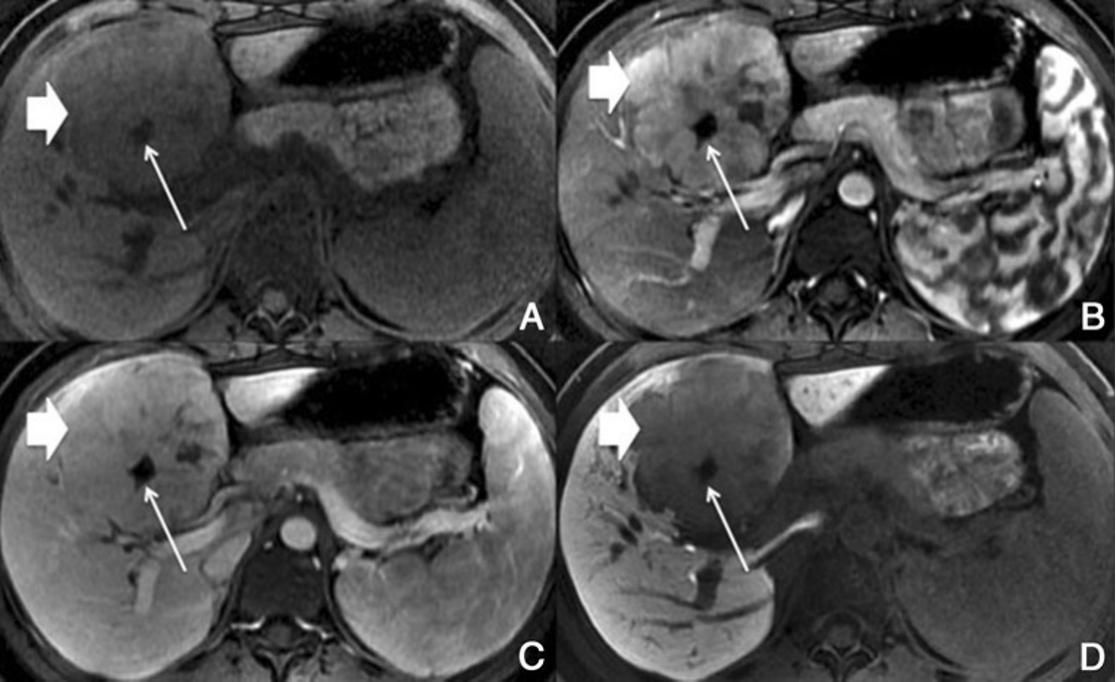
Fibrolamellar-HCC in a 34-year-old man without liver disease. **a** Pre-contrast MRI, **b** arterial phase, **c** PV phase, **d** HCP. Big nodular lesion in the left lobe isointense in pre-contrast imaging, with arterial enhancement, washout in the PV phase and no uptake of Gd-EOB-DTPA in the HCP (*thick arrow*). The central scar (*thin arrow*) remains hypointense in all sequences

There is usually early heterogeneous contrast enhancement, which fades on subsequent images. The scar has minimal or no enhancement on contrast-enhanced images, being usually hypointense relative to the remainder of the tumour (Fig. 9).

##### Imaging with Gd-EOB-DTPA

In hepatocellular phase some viable areas of the tumour may show some uptake of Gd-EOB-DTPA. The scar remains hypointense [21].

##### Differential diagnosis

Hypointensity of the scar on T2-weighted images and the hypointensity in the HCP distinguish fibrolamellar HCC from a FNH. It can be distinguished from HCC due to the presence of a scar, calcifications, larger size and occurrence in younger patients with no cirrhosis.

##### Potential pitfalls

The absence of a scar and the uptake of Gd-EOB-DTPA in the HCP may be confounders.

#### Nodular perfusional alterations versus wedge-shaped perfusional alterations (transient hepatic intensity differences [THIDs])

##### Overview

These perfusional alterations are phenomenon characterised by a patchy wedge-shaped area of enhancement involving a hepatic subsegment or by an arterial enhancing focal lesion that fades in the venous phase.

##### Unenhanced MRI features

Generally, there are no alterations in morphology or signal intensity.

##### Dynamic imaging with extracellular contrast agent

**Fig. 10:**
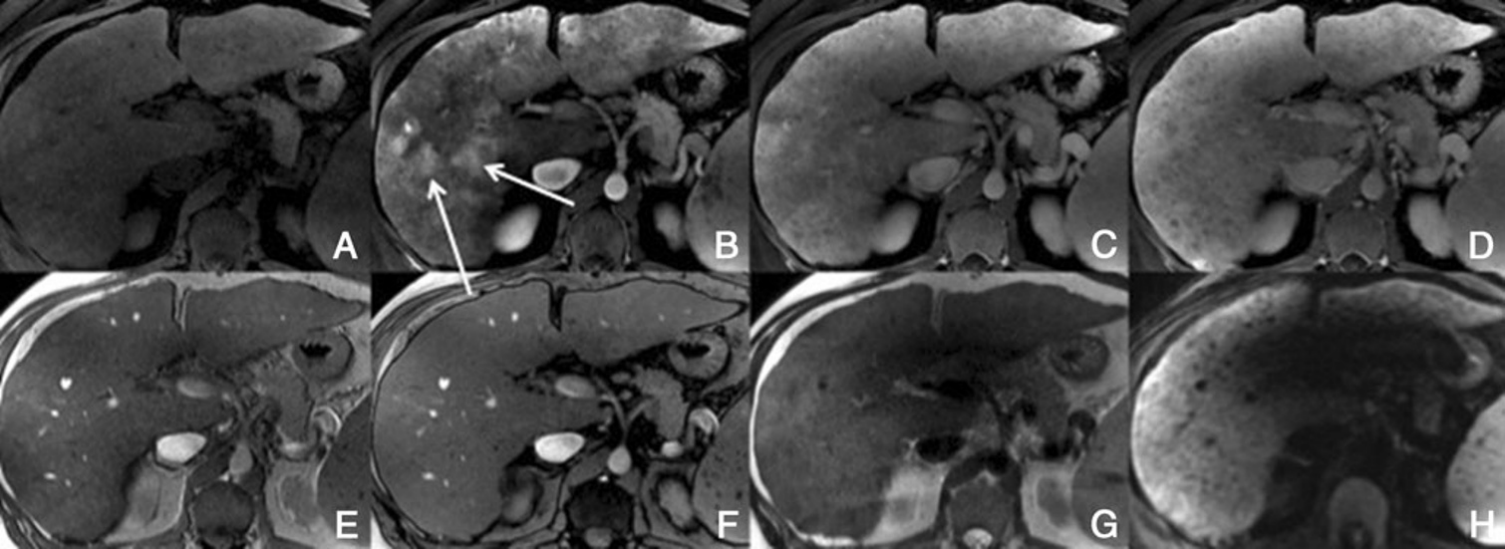
THIDs in a 38-year-old man with chronic liver disease. **a** Pre-contrast MRI, **b** arterial phase, **c** 2 min, **d** 3 min, **e** IP, **f** OP, **g** T2, **h** DWI B500. Heterogeneous liver with nodular contour. Fluffy hyperintense areas in the arterial phase (*arrows*), not seen in the other sequences

**Fig. 11:**
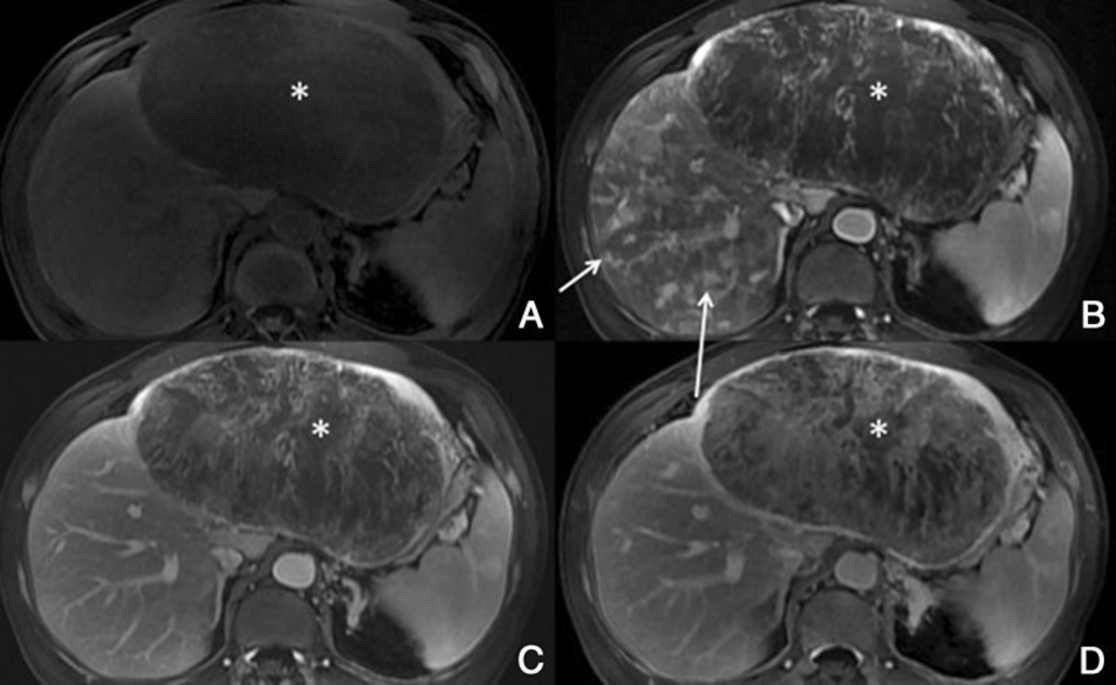
Multiple THIDs and a big HCC in a 47-year-old man with chronic liver disease. **a** Pre-contrast MRI, **b** arterial phase, **c** PV phase, **d** delayed phase. Big nodular lesion in the left lobe hypointense in pre-contrast imaging, with arterial enhancement, internal arteries, washout in the PV phase and heterogeneous uptake of Gd-EOB-DTPA in the HCP (*). There also seen multiple nodulariform hyperintense areas in the arterial phase (*arrows*), not seen in the other sequences—THIDs

This finding is seen immediately after contrast material administration, with fading on subsequent images. They are thought to represent an imbalance between the hepatic arterial and portal venous supply, caused by increased hepatic arterial blood flow in the presence of portal vein obstruction (Figs. 10 and 11). Typically, transiently increased segmental perfusion enhancement indicates that the portal venous supply is compromised due to compression or thrombosis.

##### Imaging with Gd-EOB-DTPA

No alterations in the hepatobiliary phase, due to intact hepatocyte function of these lesions, make it possible to differentiate from small HCCs [22, 23].

##### Differential diagnosis

Small HCCs and hypervascular metastases are possible diagnosis. Usually the absence of a defect in the HCP makes the diagnosis of THID.

##### Potential pitfalls

Rarely, vascular alterations are associated with dysfunction of the hepatocytes, which reveal a defect in the HCP, complicating the diagnosis.

#### Focal nodular hyperplasia (FNH)

##### Overview

FNH is the second most common benign tumour of the liver, after hepatic haemangiomas. FNH typically occurs as a solitary lesion in young female patients. It consists of aggregates of hepatocytes and is thought to be secondary to a proliferative response of hepatocytes, secondary to an underlying vascular malformation. Biliary structures proliferate without connection to the adjacent biliary tree. Kupffer cells are present, often in greater amounts than in the surrounding normal liver parenchyma. The central scar in FNH is not a true scar, but represents a congeries of blood vessels, bile ducts and sometimes a focal area of cirrhosis. About 20% of FNH cases are classified as non-classic. These non-classic FNH subtypes lack the findings of nodular architecture or malformed vessels but always have bile duct proliferation.

##### Unenhanced MRI features

The lesion varies from mildly hypointense to isointense on T1-weighted MR images and from mildly hyperintense to isointense on T2-weighted images. The “central scar” is characteristically hyperintense on T2-weighted images. The hyperintensity of the lesion on T2-weighted images may be related to the presence of vascular channels or oedema throughout the lesion.

##### Dynamic imaging with extracellular contrast agent

FNH is perfused by the hepatic arterial system and shows marked, nearly uniform arterial-phase enhancement. The degree of lesion enhancement lessens on subsequent contrast-enhanced images, with lesion signal intensity approaching that of the surrounding liver parenchyma. The central scar has low signal intensity on early phase contrast-enhanced images but gradually enhances to become hyperintense relative to the rest of the lesion on delayed phase images [24, 25]. The capsule is usually absent.

##### Imaging with Gd-EOB-DTPA

**Fig. 12:**
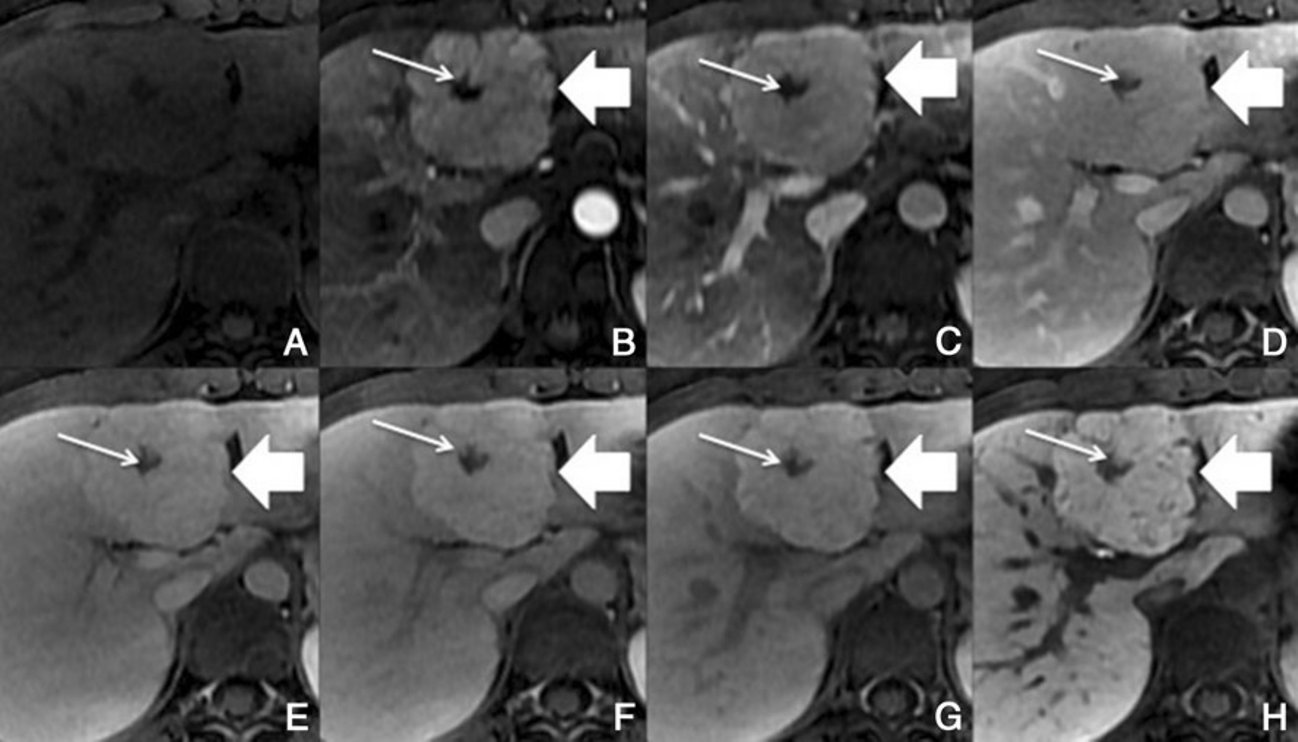
FNH in a 37-year-old woman with no liver disease. **a** Pre-contrast MRI, **b, c** arterial phase, **d** 3 min, **e** 5 min, **f** 8 min, **g** 10 min, **h** HCP. Big nodular lesion (*thick arrows*), with lobulated contours isointense in pre-contrast imaging, with arterial enhancement, washout in the PV phase and uptake of Gd-EOB-DTPA in the HCP—note the fine reticular architecture typical of a FNH. The central scar (*thin arrow*) remains hypointense in all sequences

With hepatobiliary-specific agents, it shows homogenous or heterogeneous enhancement in the hepatocellular phase, as it lacks a well-formed bile canalicular system for normal excretion. It becomes isointense to hyperintense to the normal liver, allowing the differential diagnosis [8, 26]. More than the enhancement itself, the hepatocellular phase also permits the evaluation of the internal architecture (reticulation or lace-like appearance), which provides more certainty in the diagnosis (Fig. 12).

##### Differential diagnosis

Adenoma and fibrolamellar HCC. The isointensity to hyperintensity in the HCP and the imaging features of the central scar of FNH are usually helpful in the differential diagnosis. The adenoma does not present a central scar and is hypointense in the HCP. The F-HCC is usually larger, more heterogeneous and its scar is hypointense in every sequence.

Also, the non-classic forms often present a diagnostic challenge in imaging: e.g. telangiectatic FNH has been shown to demonstrate atypical imaging features, including lack of a central scar, high signal intensity on T1- and T2-weighted images and persistent enhancement on delayed phase images [26].

##### Potential pitfalls

About 12% of FNH can show atypical imaging features on Gd-EOB-DTPA enhanced MRI, such as the absence of a scar or an eccentric one, or washout in the venous phase.

#### Adenoma

##### Overview

Hepatic adenomas are rare benign tumours of the liver. On histopathological analysis, hepatic adenomas contain well-differentiated hepatocytes lacking bile ducts or portal tracts. The majority of adenomas are solitary (80%) and typically occur in female patients (90%). Predisposing factors to adenoma formation include oral contraceptive use in female patients, anabolic steroid use in male patients and glycogen storage disease [27].

##### Unenhanced MRI features

They are generally well circumscribed, without contour lobulation and have variable degrees of haemorrhage, necrosis, fat and, rarely, calcification. At MR imaging, adenomas are typically hyperintense to isointense on T1-weighted images and slightly hyperintense on T2-weighted images.

##### Dynamic imaging with extracellular contrast agent

After administration of contrast material, adenomas typically demonstrate moderate enhancement on arterial phase images; they may show washout and enhancement similar to that of surrounding liver parenchyma on portal venous and delayed phase images [28].

##### Imaging with Gd-EOB-DTPA

**Fig. 13:**
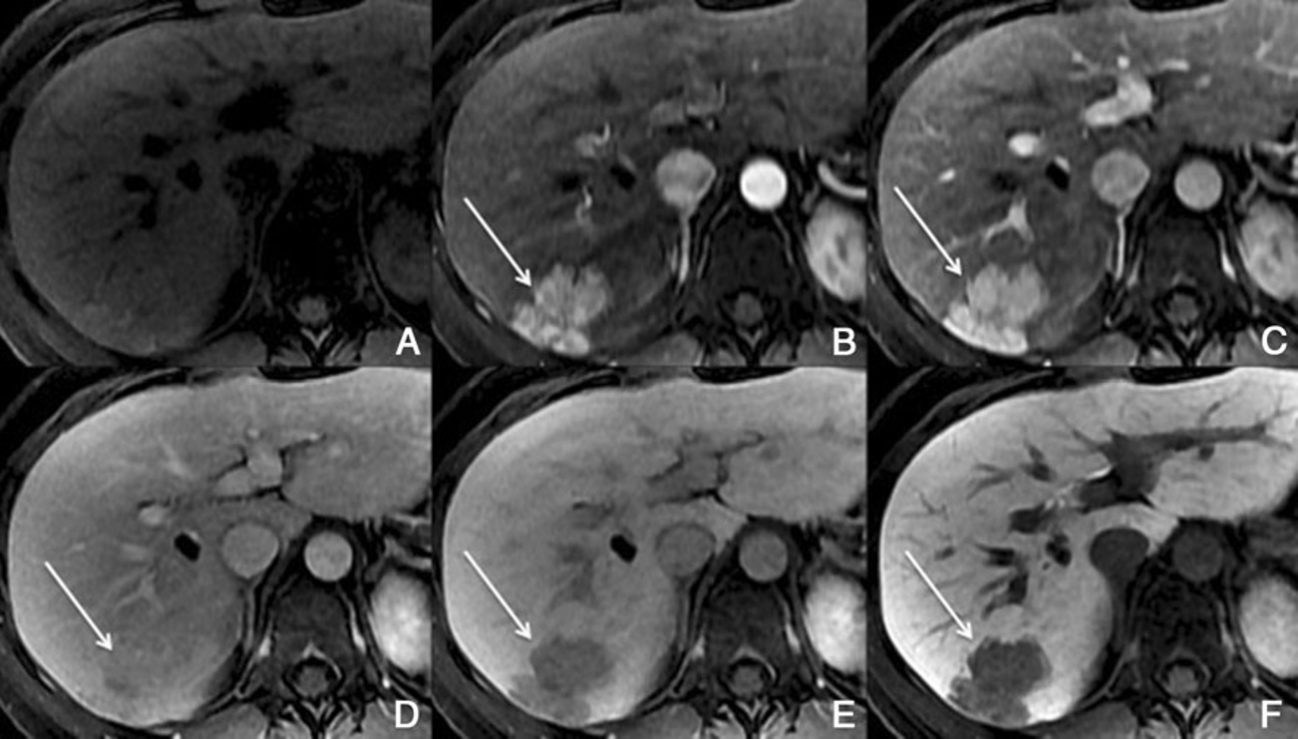
Liver adenoma in a 35-year-old woman on oral contraceptives, with no liver disease. **a** Pre-contrast MRI, **b, c** arterial phase, **d** 3 min, **e** 5 min, **f** HCP. Lobulated lesion in the right lobe isointense in pre-contrast imaging, with arterial enhancement, internal arteries and no uptake of Gd-EOB-DTPA in the HCP (*arrow*)

Usually no enhancement of hepatic adenoma is seen in the hepatocellular phase because of absent or strongly reduced hepatocellular uptake of Gd-EOB-DTPA (Fig. 13). However, peripheral or faint diffuse enhancement has been reported in some cases due to some hepatocytes maintaining the capability of uptake and excretion in the HCP.

##### Differential diagnosis

FNH and HCC.

The age and gender of the patient, occurring in a non-cirrhotic liver (as opposed to HCC), the absence of a scar or the enhancement pattern, usually helps in the diagnosis, since FNH is isointense to hyperintense in the HCP, while the adenoma is typically hypointense.

##### Potential pitfalls

The adenoma may show faint enhancement in the HCP, which is a confounder, but the other imaging characteristics usually permit the diagnosis.

#### Focal hypereosinophilic infiltration (FEI)

##### Overview

FEI of the liver is a focal hepatic lesion caused by eosinophil-related tissue damage. It is associated with various eosinophilia-related conditions such as parasitic infections, allergic reactions, hypereosinophilic syndrome and tumours.

##### Unenhanced MRI features

They have an indistinct margin and a non-spherical shape [29, 30]. On T1- and T2-weighted images, visible focal eosinophilic infiltrations and eosinophilic abscesses exhibit low and high signal intensities, respectively.

##### Dynamic imaging with extracellular contrast agent

The dynamic imaging is characterised by poor enhancement on the arterial phase with homogeneous enhancement on portal venous phase images.

##### Imaging with Gd-EOB-DTPA

**Fig. 14:**
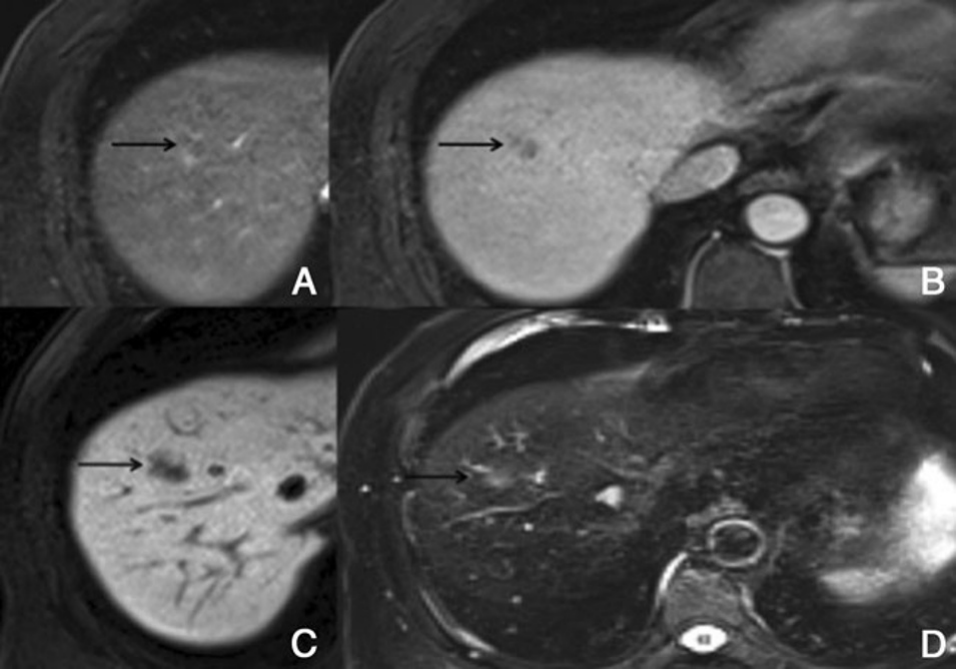
Focal hypereosinophilic necrosis nodule in a 55-year-old man with no liver disease. **a** Arterial phase, **b** PV phase, **c** HCP, **d** T2-weighted. Small non-spherical lesion with ill-defined margins in the right lobe with poor enhancement in the arterial and PV phase, mixed hypointensity on the HCP and slightly hyperintense in T2-weighted (*black arrows*). This lesion was biopsy proven to be a lesion of focal eosinophilic infiltration

They show mixed hypointensity, irregular margins and non-spherical shapes in hepatocellular phase (Fig. 14).

##### Differential diagnosis

Metastases and abscesses. The non-spherical shape and irregular margins favour FEI.

##### Potential pitfalls

The differential diagnosis from metastases in a patient with known primary malignancy is still challenging.

#### Confluent fibrosis

##### Overview

Focal fibrosis occurs in the process of hepatic parenchyma collapse and its replacement with focal fibrotic masses, in the context of cirrhosis. It is usually located in the anterior and medial segments of the liver and has a wedge-shaped appearance. In some patients the entire segment is involved.

##### Unenhanced MRI features

Fibrosis can appear as an area of low signal intensity on T1-weighted MR images and high signal intensity on T2-weighted images. It shows a typical geographic pattern of involvement, associated to retraction of the liver capsule.

##### Dynamic imaging with extracellular contrast agent

It usually demonstrates delayed enhancement on contrast-enhanced images.

##### Imaging with Gd-EOB-DTPA

**Fig. 15:**
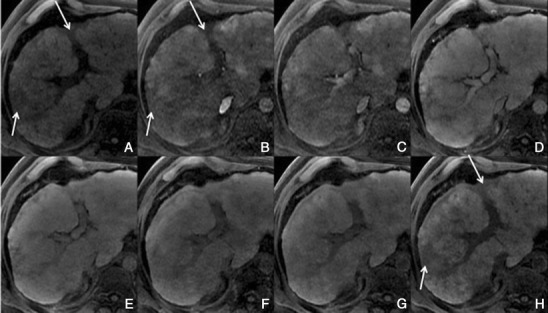
Confluent fibrosis in a 56-year-old man with cirrhosis. **a** Pre-contrast, **b, c** arterial phase, **d** PV phase, **e** 3 min, **f** 5 min, **g** 8 min, **h** HCP. Wedge-shaped ill-defined areas associated with capsular retraction, with decreased enhancement in the dynamic phases and with no uptake of Gd-EOB-DTPA in the HCP (*arrows*)

It shows low signal intensity due to decreased hepatic function from fibrosis (Fig. 15).

##### Differential diagnosis

Infiltrative HCC and cholangiocarcinoma. Confluent fibrosis can be differentiated from infiltrative tumours on the basis of typical geographic appearance, location, no adjacent vascular invasion and no surrounding ductal dilatation.

##### Potential pitfalls

Contrary to delayed enhancement of fibrosis on gadolinium-enhanced MRI, confluent hepatic fibrosis demonstrates hypointense areas due to decreased or absent hepatocellular uptake of Gd-EOB-DTPA.

### Lesions in which Gd-EOB-DTPA has pitfalls and thus requires careful interpretation

#### Cavernous haemangioma

##### Overview

Cavernous haemangiomas represent the most common primary liver tumour and consist of multiple large, blood-filled vascular channels. Typically, haemangiomas are solitary, although multiplicity is not uncommon. Although they are typically small, giant cavernous haemangiomas may range in size (greater than 20 cm). Haemangiomas are associated with several clinical syndromes including Klippel-Trenaunay-Weber syndrome, Osler-Rendu-Weber disease, and von Hippel–Lindau disease.

##### Unenhanced MRI features

On MR imaging, hepatic haemangiomas typically demonstrate low signal intensity on T1-weighted images and marked high signal intensity on T2-weighted images.

##### Dynamic imaging with extracellular contrast agent

On enhanced MRI, there is a typical peripheral nodular enhancement with centripetal progression, resulting in diffuse high signal intensity on delayed phase images. Less commonly, flash filling of an entire small haemangioma or enhancement of small portions of the lesion, on arterial phase images, may be seen. Larger lesions may not demonstrate uniform enhancement on delayed phase images, probably due to areas of thrombosis or insufficient delay time. Central scars may be seen in hepatic haemangiomas.

##### Imaging with Gd-EOB-DTPA

**Fig. 16:**
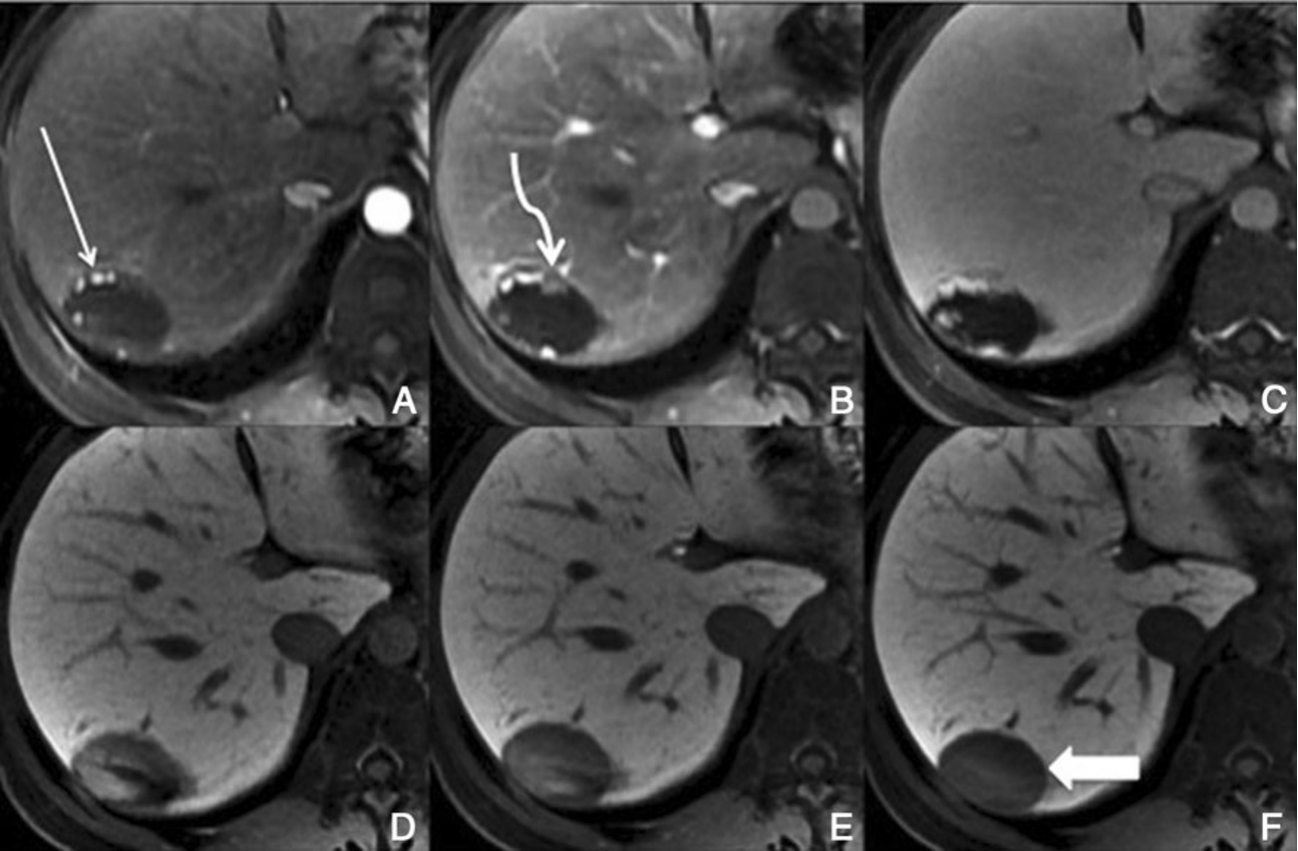
Haemangioma in a 39-year-old woman with no liver disease. **a, b** Arterial phase, **c** PV phase, **d** 3 min, **e** 5 min, **f** HCP. Right lobe hypointense lesion with arterial nodular peripherical enhancement (*thin arrow*) with centripetal progression. Note that the hepatocellular uptake starts immediately (*curved arrow*). The intralesional enhancement follows the blood pool. In the HCP the lesion is hypointense because of rapid uptake of Gd-EOB-DTPA by the surrounding normal liver during equilibrium phase (*thick arrow*)

With this contrast, the enhancement of a haemangioma tends to follow the blood vessels. Hepatic haemangiomas will “pseudo washout” to become darker than the liver because of rapid uptake of the contrast agent by the surrounding normal liver during equilibrium phase (Fig. 16). However, careful analysis usually permits differential diagnosis from malignant lesions that washout, as malignant lesions will washout more rapidly than the vessels, while haemangiomas will parallel the vessels [31].

##### Differential diagnosis

Small haemangioma may mimic hypervascular metastasis. In these cases, the bright signal of haemangiomas in heavily T2-weighted sequences is helpful. Furthermore, in the dynamic phases, the intralesional degree of enhancement is similar to that of the adjacent vessels, pointing to a lesion filling with less enhanced blood due to the uptake of the hepatocytes.

##### Potential pitfalls

In patients with prolonged intravascular dwell time due to hepatic or renal insufficiency, haemangiomas may appear isointense to the liver in the hepatocellular phase rather than hypointense. The key is parallelism to the blood pool.

#### Focal fat/focal sparing

##### Overview

Focal fatty deposition and focal fatty sparing may manifest as a “pseudolesion”. Clues to this diagnosis are its more common occurrence in the following areas of the liver: the medial aspect of the left lobe, along the falciform ligament, round the gallbladder fossa and within the central aspect of segment IV, adjacent to the porta hepatis. In addition, focal fatty infiltration is not seen to exert mass effect and vessels are seen to pass through these areas unimpeded.

##### Unenhanced MRI features

MR imaging provides a useful method for characterising suspected geographic fatty infiltration by using in-phase and opposed-phase gradient-echo T1-weighted sequences. The relative increase in intravoxel fat in areas of focal fatty infiltration demonstrates decreased signal intensity on the opposed-phase images in comparison to the in-phase images. On MR imaging, areas of fatty sparing may be identified due to the lack of decrease in signal intensity on opposed-phase gradient-echo T1-weighted images and appear hyperintense relative to surrounding steatosis. Focal fat often appears dark on opposed-phase images, while focal sparing often appears bright [32].

##### Dynamic imaging with extracellular contrast agent

Focal fat deposition and fat sparing areas show similar degree of enhancement to surrounding hepatic parenchyma.

##### Imaging with Gd-EOB-DTPA

**Fig. 17:**
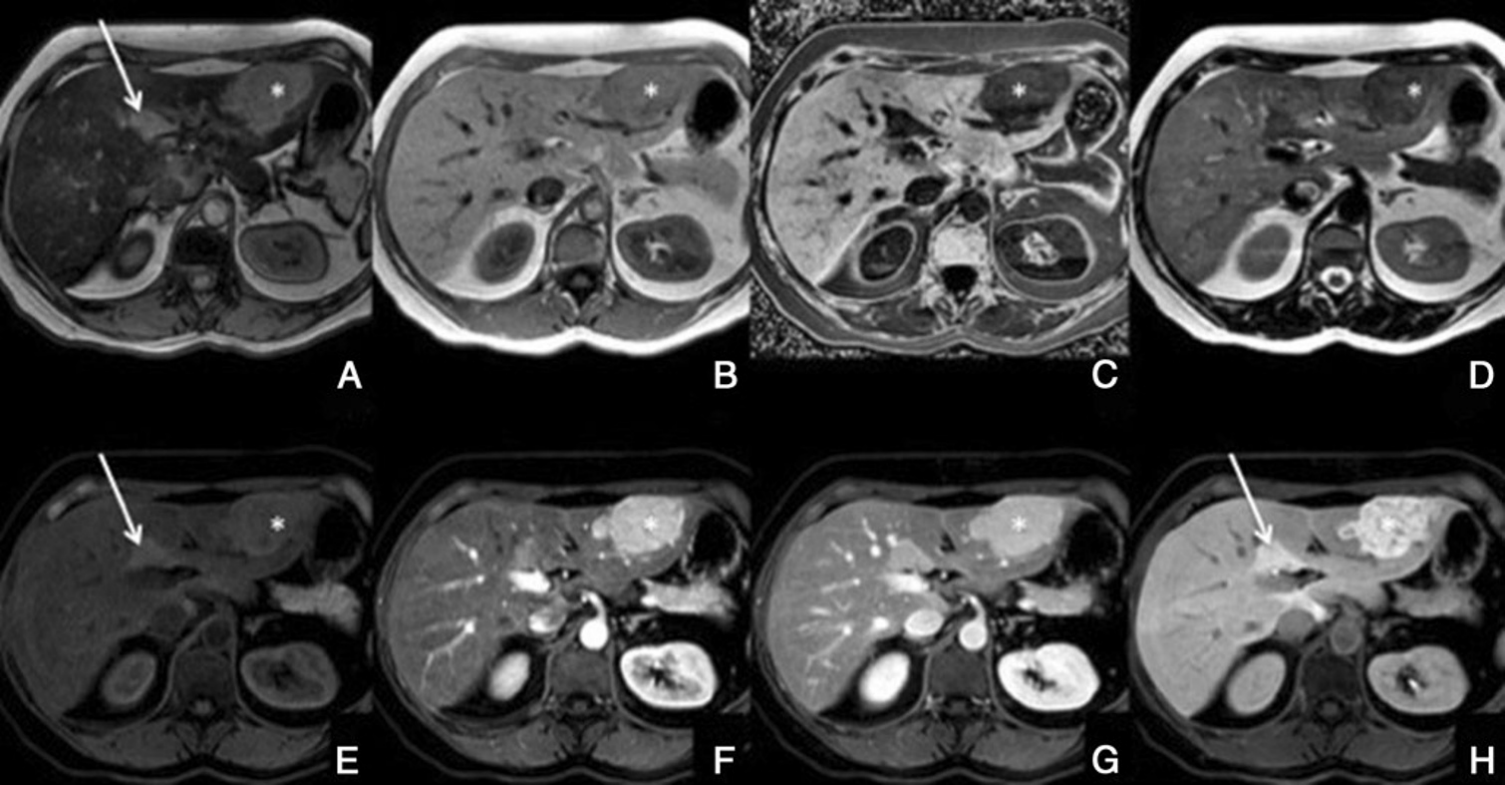
Focal fat sparing area and a FNH, in a 36-year-old woman with no liver disease. **a** OP, **b** IP, **c** PDFF, **d** T2-weighted, **e** T1 fat-sat, **f** arterial phase, **g** PV phase, **h** HCP. Triangular-shaped periportal area (*arrows*) hyperintense to the rest of the liver in OP and Fat Sat imaging mimicking uptake of Gd-EOB-DTPA, corresponding to a fat spared area. This is confirmed in the fat-fraction map. Of note, is a lesion (*) compatible with a FNH

Areas of focal fat sparing area can be shown as mildly increased signal intensity on the hepatobiliary phase because the signal intensity of the focal fat sparing area is already higher on the unenhanced fat saturated sequence (Fig. 17).

##### Differential diagnosis

Focal hepatic lesion and lipoma. The typical locations and the T2 findings usually permit the diagnosis, since these pseudo-lesions are invisible on that sequence.

##### Potential pitfalls

Areas of focal fat sparing are enhanced because they have functional hepatocytes and these can appear as real focal lesions in the dynamic phases. However, the other imaging findings described permit the diagnosis.

#### Intrahepatic cholangiocarcinoma

##### Overview

Cholangiocarcinoma is the most common biliary tumour and the second most common primary malignant hepatic tumour in adults. Risk factors for the development of cholangiocarcinoma include primary sclerosing cholangitis (PSC), familial polyposis, choledochal cyst, biliary papillomatosis and clonorchiasis.

##### Unenhanced MRI features

Intrahepatic cholangiocarcinoma may be mass forming or peribiliar infiltrative. They are usually hypointense on T1-weighted MR images and hyperintense on T2-weighted images. Associated findings may include adjacent biliary ductal dilatation, capsular retraction, satellite nodules, vascular encasement and hepatolithiasis [32–34].

##### Dynamic imaging with extracellular contrast agent

On contrast-enhanced images, it demonstrates initial peripheral enhancement with concentric internal filling on delayed-phase images.

##### Imaging with Gd-EOB-DTPA

**Fig. 18:**
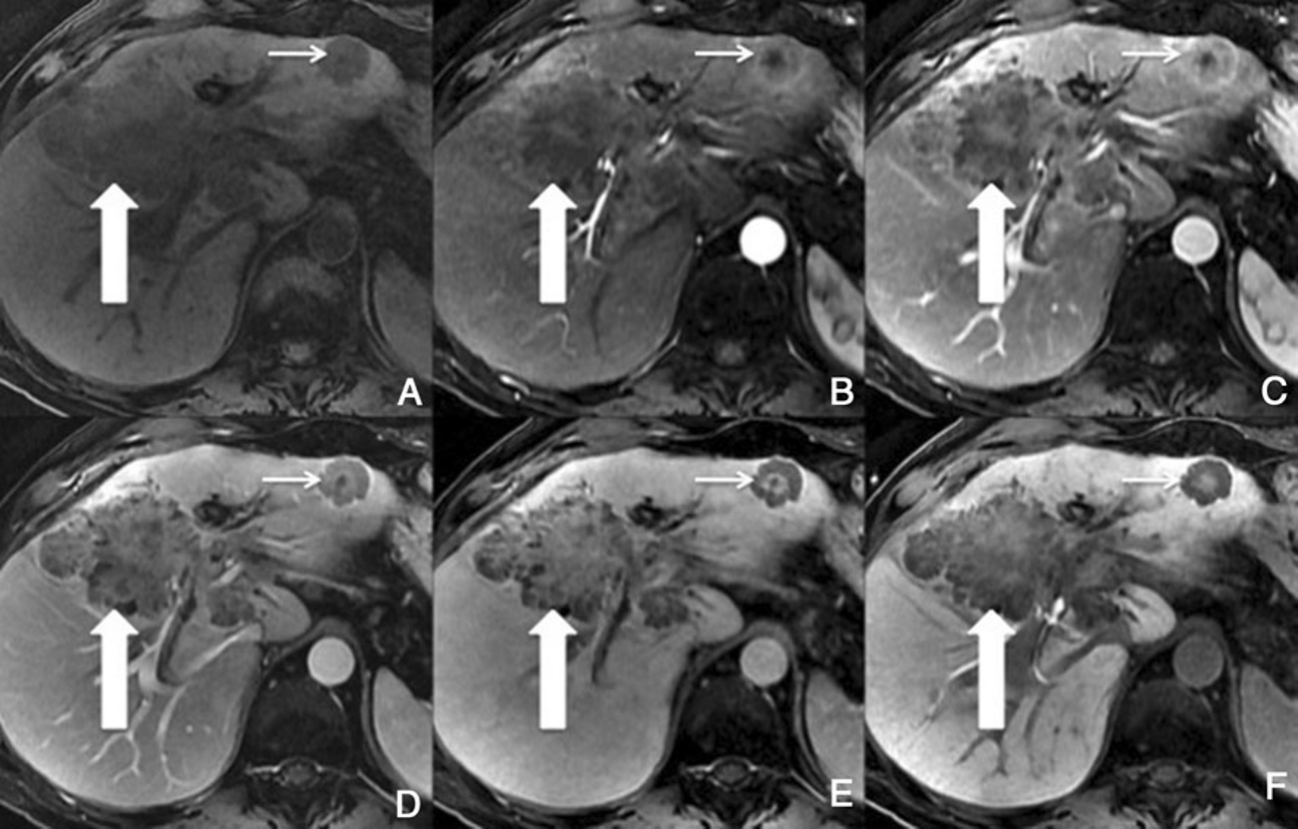
Cholangiocarcinoma with a satellite lesion, in a 62-year-old man. **a** Pre-contrast, **b, c** arterial, **d** 3 min, **e** 5 min, **f** HCP. Large mass-forming lesion, irregular contour, with mild heterogeneous enhancement in the dynamic phases, with no uptake of Gd-EOB-DTPA in the HCP (*thick arrow*) There is another lesion (*thin arrow*) in the left lobe with similar contrast uptake proprieties and a necrotic centre, compatible with a metastasis

Usually no enhancement is seen in the hepatocellular phase due to lack of hepatocytes (Fig. 18).

##### Differential diagnosis

HCC and cholangitis. The delayed enhancement and the typical hilar location point to cholangiocarcinoma.

##### Potential pitfalls

Delayed enhancement of cholangiocarcinoma is one of the characteristic findings when using extracellular agents, due to its abundant fibrosis. However, the tumour shows low signal intensity on delayed and hepatobiliary phases of Gd-EOB-DTPA, due to strong enhancement of the surrounding liver parenchyma.

#### Hypervascular metastases

##### Overview

Islet cell tumours, breast cancer, melanoma, thyroid cancer and carcinoid tumour are among the most common primary tumours that lead to hypervascular hepatic metastases.

##### Unenhanced MRI features

These metastatic lesions usually demonstrate low signal intensity on T1-weighted MR images and are iso to hyperintense on T2-weighted images.

##### Dynamic imaging with extracellular contrast agent

Hypervascular metastases are best seen during the arterial phase of enhancement. Small hypervascular metastases usually present with homogeneous enhancement, whereas larger lesions appear heterogeneous or show an enhancing peripheral rim surrounding the necrosis. Frequently, centripetal contrast enhancement is observed, as well as irregular or peripheral washout. Hypervascular metastatic lesions may show a target appearance on delayed phase, with the rim appearing hypointense relative to the centre. This centripetal progression of enhancement, with simultaneous peripheral washout, is specific for malignancy.

##### Imaging with Gd-EOB-DTPA

Generally no enhancement is seen in the hepatocellular phase.

##### Differential diagnosis

HCC, THID, adenoma and FNH. The presence of multiple lesions in a non-cirrhotic liver, in a patient with a known malignancy and a defect on the HCP point to metastases.

##### Potential pitfalls

**Fig. 19:**
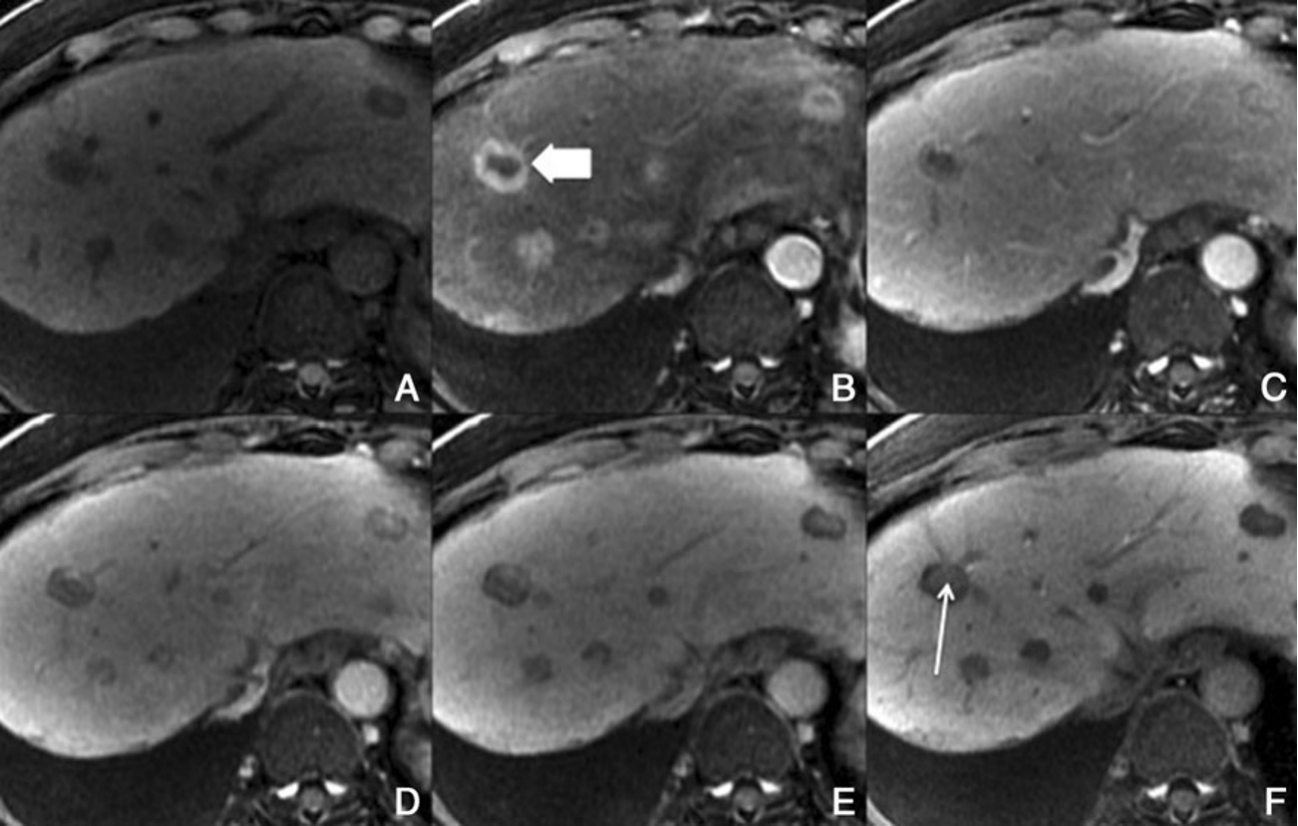
Multiple hypervascular metastases, in a 39-year-old woman with breast cancer. **a** Pre-contrast, **b** arterial phase, **c** PVP, **d** 3 min, **e** 5 min, **f** HCP. Multiple nodular lesions are seen, hyperenhancing in the arterial phase, with washout in the PVP, some with a hypointense necrotic centre (*solid arrow*). In the HCP, some of these lesions paradoxically uptake Gd-EOB-DTPA in the necrotic centre (*thin arrow*)

In the centre of metastatic lesions, there may be pooling of Gd-EOB-DTPA in the extracellular matrix of the lesion, giving the misguiding appearance of uptake in the hepatobiliary phase (Fig. 19).

#### Hypovascular metastases

##### Overview

Colon, lung, prostate, gastric and transitional cell carcinomas are the most common primary tumours with hypovascular metastases to the liver.

##### Unenhanced MRI features

These metastatic lesions usually demonstrate low signal intensity on T1-weighted MR images and are iso to hyperintense on T2-weighted images.

##### Dynamic imaging with extracellular contrast agent

**Fig. 20:**
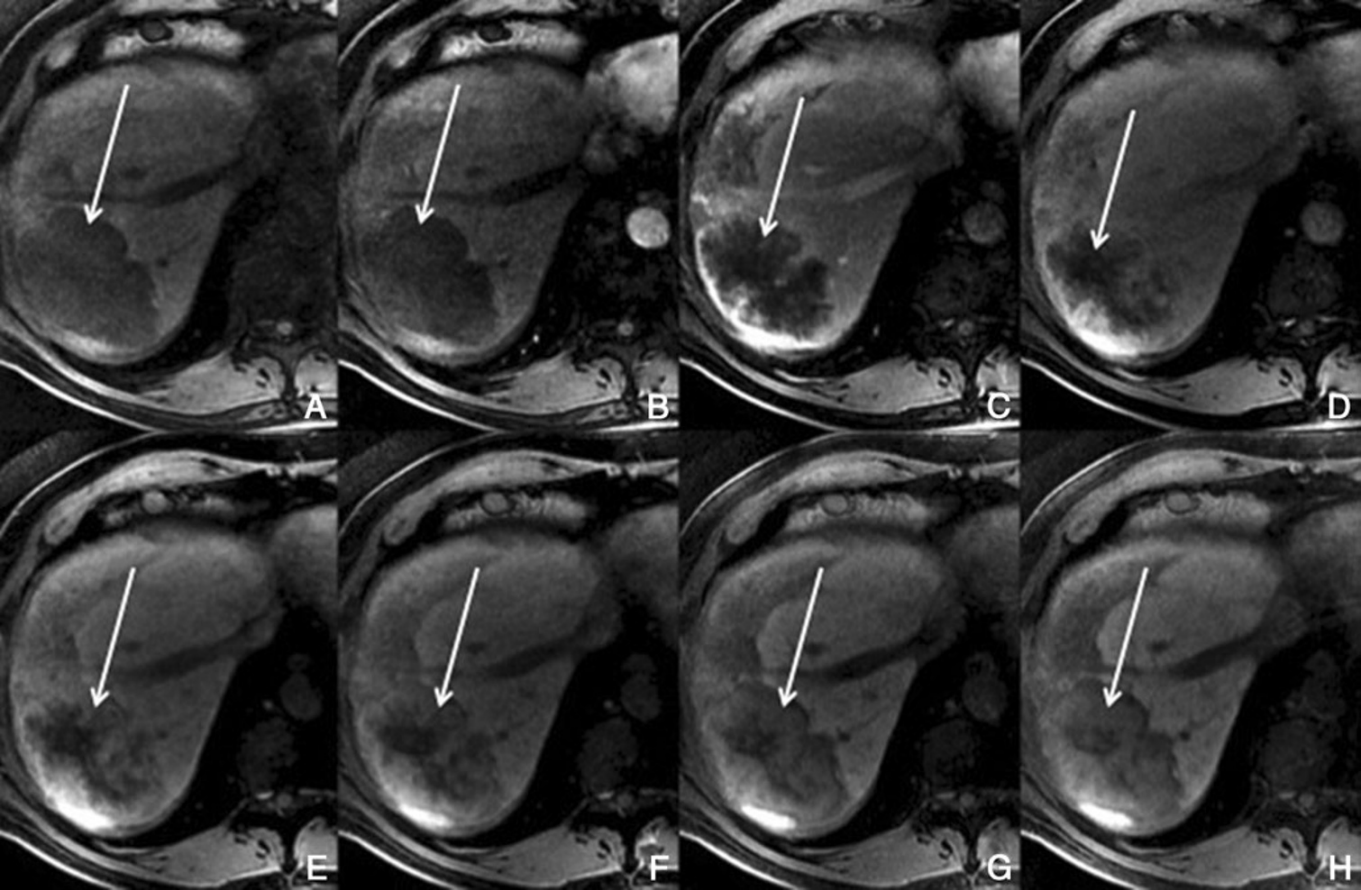
Multiple hypovascular metastases, in a 60-year-old man with an NE tumour. **a** Pre-contrast, **b, c** arterial, **d** 3 min, **e** 5 min, **f** 8 min, **g** 10 min, **h** HCP. Multiple hypointense nodular lesions, with no significant enhancement in the dynamic phases, one of large dimensions in the right lobe (*arrows*), with progressive central pooling of Gd-EOB-DTPA, more evident in the HCP

The ring-like enhancement surrounding liver metastases is typically seen during the hepatic arterial phase. In the portal and delayed phase, the metastases often show washout in the outer parts and a progressive enhancement toward the centre of the lesions (Fig. 20).

##### Imaging with Gd-EOB-DTPA

No enhancement is seen in the hepatocellular phase.

##### Differential diagnosis

HCC, cholangiocarcinoma and focal eosinophilic infiltration. Multiple lesions, a known primary malignant tumour and a non-cirrhotic liver, point to the diagnosis. The pattern of enhancement and the spherical shape are other helpful findings.

##### Potential pitfalls

As mentioned earlier, with Gd-EOB-DTPA, the differential diagnosis can be difficult.

#### Foreign body reaction

##### Overview

Various foreign bodies introduced into the human organism during surgery or trauma, as well as exposure to some chemical substances, may cause a granulomatous reaction.

##### Unenhanced MRI features

Imaging features are usually hypointensity in T1-weighted images and hyperintensity in T2-weighted images, with possible hypointense bands within the cystic cavity.

##### Dynamic imaging with extracellular contrast agent:

It shows peripheral enhancement of the mass on arterial or portal venous phase.

##### Imaging with Gd-EOB-DTPA

**Fig. 21:**
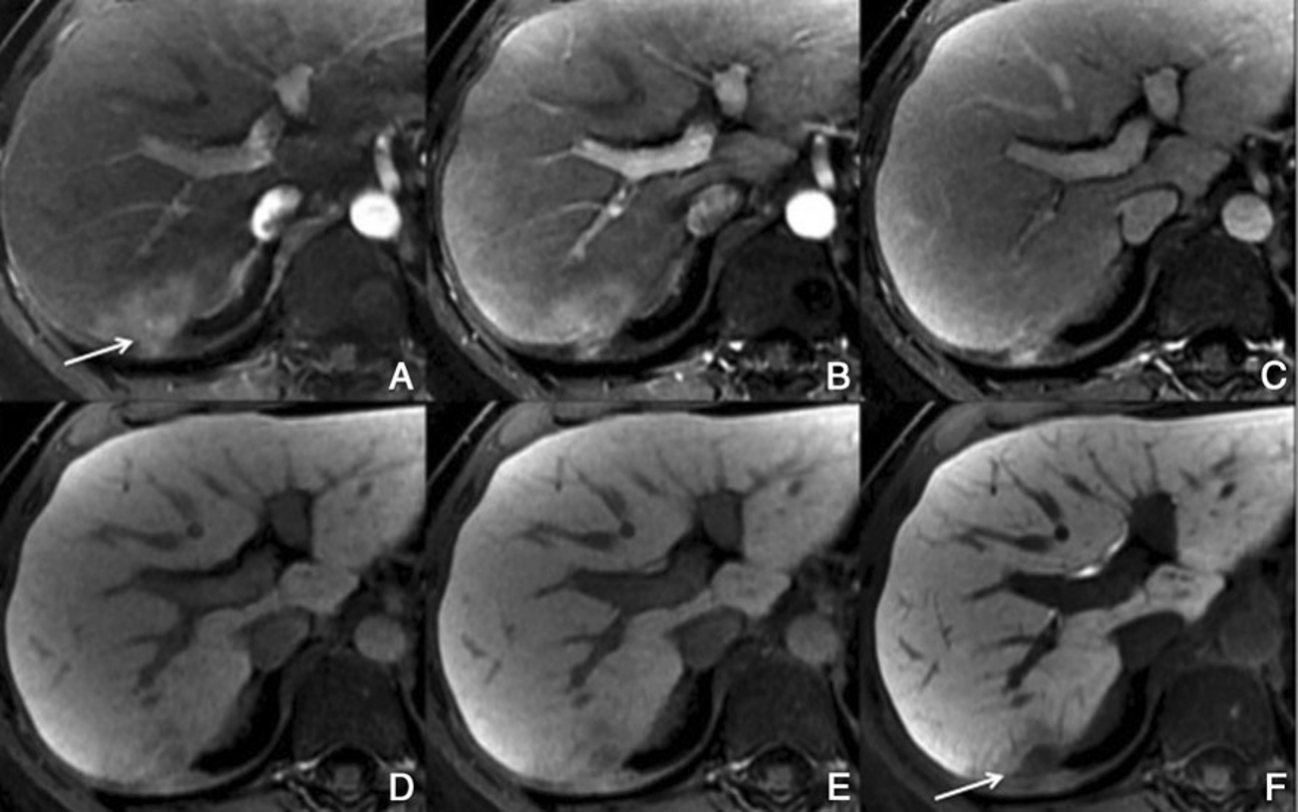
Foreign body reaction, in a 41-year-old man that suffered a car accident after a New Year’s dinner. **a, b** Arterial phase, **c** PVP, **d** 5 min, **e** 8 min, **f** HCP. In the posterior aspect of the left lobe we see a fluffy heterogeneous hyperenhancing area, that progressively washes out and gives origin to a Gd-EOB-DTPA defect (*thin arrows*). Histologically proven, after surgery, to be an inflammatory reaction to a small carrot fragment

No enhancement is seen in the hepatocellular phase (Fig. 21).

##### Differential diagnosis

Metastatic disease. It could be considered in patients with a perihepatic lesion with previous history of trauma or surgery.

##### Potential pitfalls

A foreign body can gradually move from a subcapsular to an intraparenchymal position.

### Lesions in which the lesion behaviour is equivalent to that of when using an ECF contrast agent

**Fig. 22:**
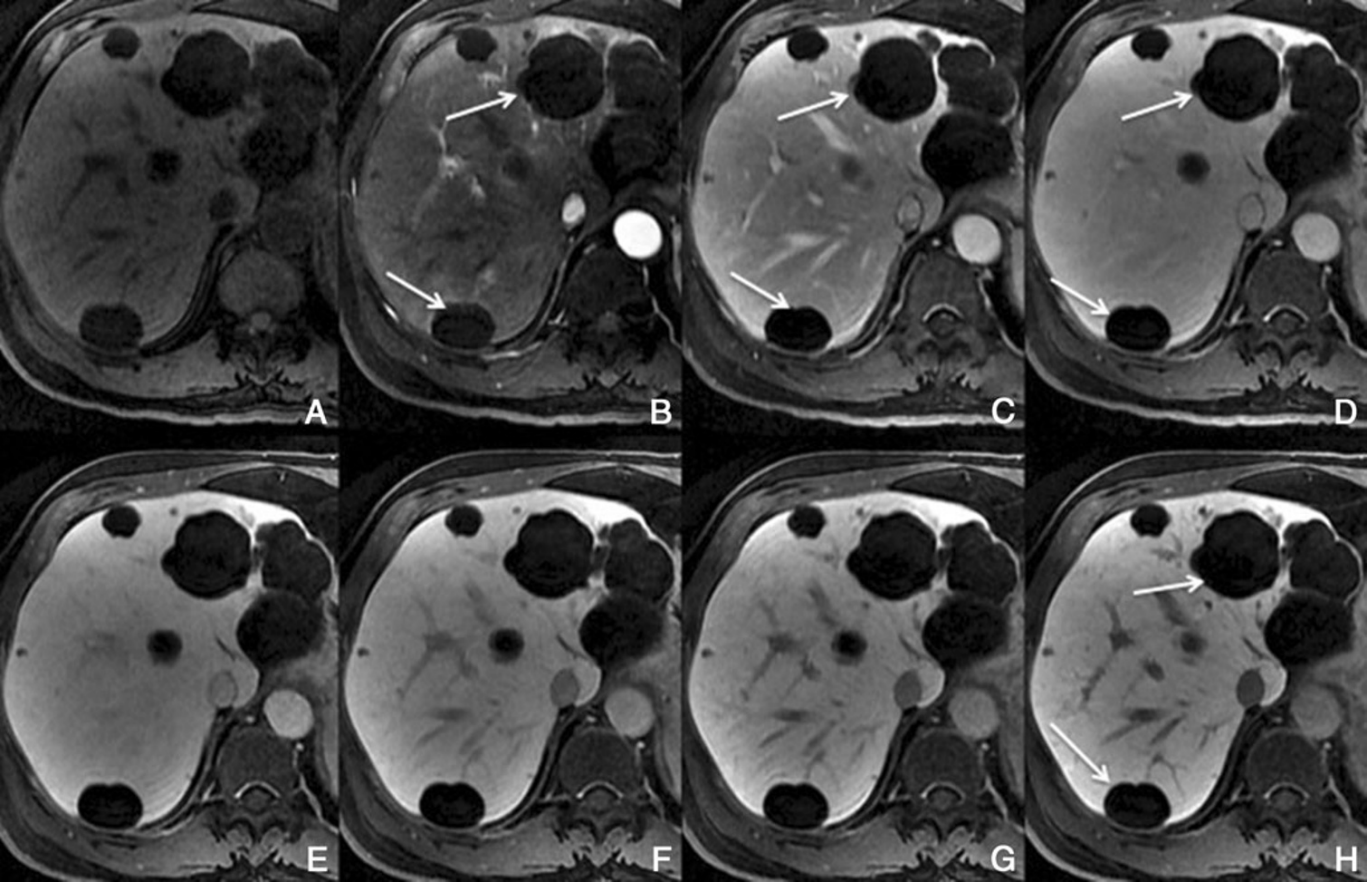
Multiple hepatic cysts, in a 47-year-old woman, with no liver disease. **a** Pre-contrast, **b** arterial, **c** PVP, **d** 3 min, **e** 5 min, **f** 8 min, **g** 10 min, **h** HCP. Multiple rounded hypointense lesions (*arrows*), with no enhancement during all the phases. These lesions were also markedly hyperintense in T2-weighted imaging (not shown)

**Fig. 23:**
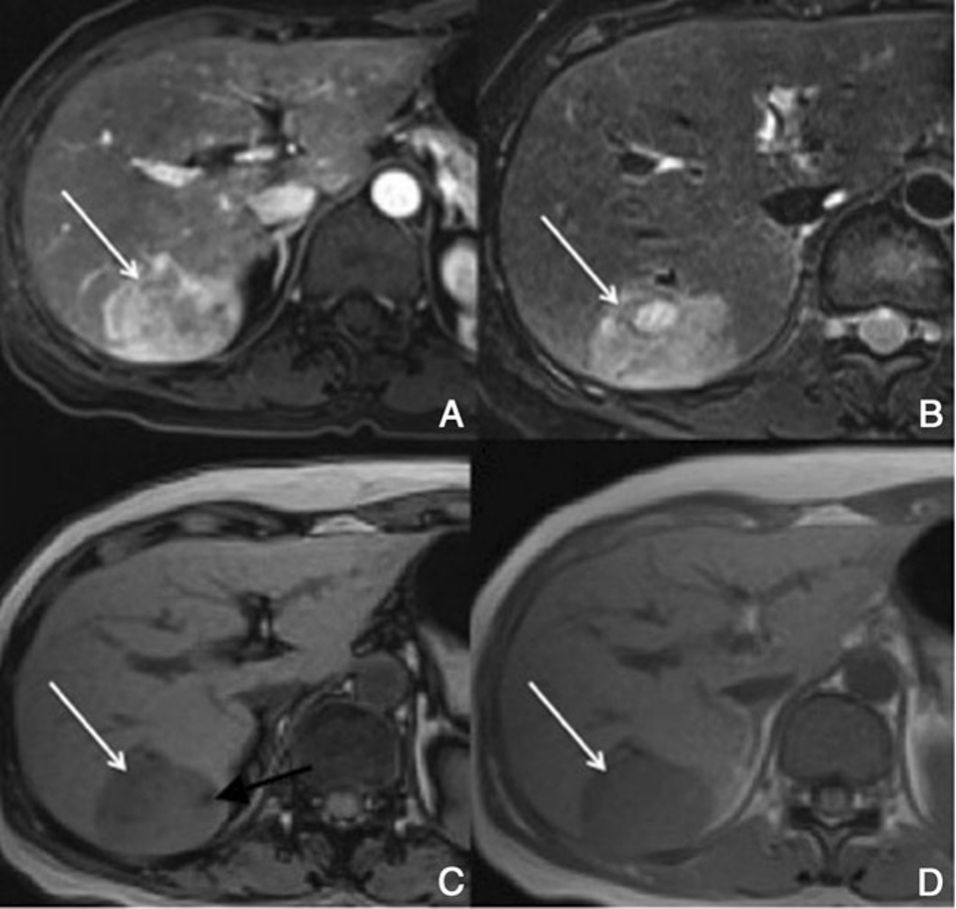
Angiomyolipoma in a 40-year-old man with a history of renal lesions. **a** Arterial phase, **b** T2 fat sat, **c** OP, **d** IP. Large mass-forming lesion, irregular contour, with mild heterogeneous enhancement in the arterial phase (*arrow*). It loses internal signal in OP, indicating the presence of intracellular fat (*black arrow*). These were histologically proven to be an angiomyolipoma

**Fig. 24:**
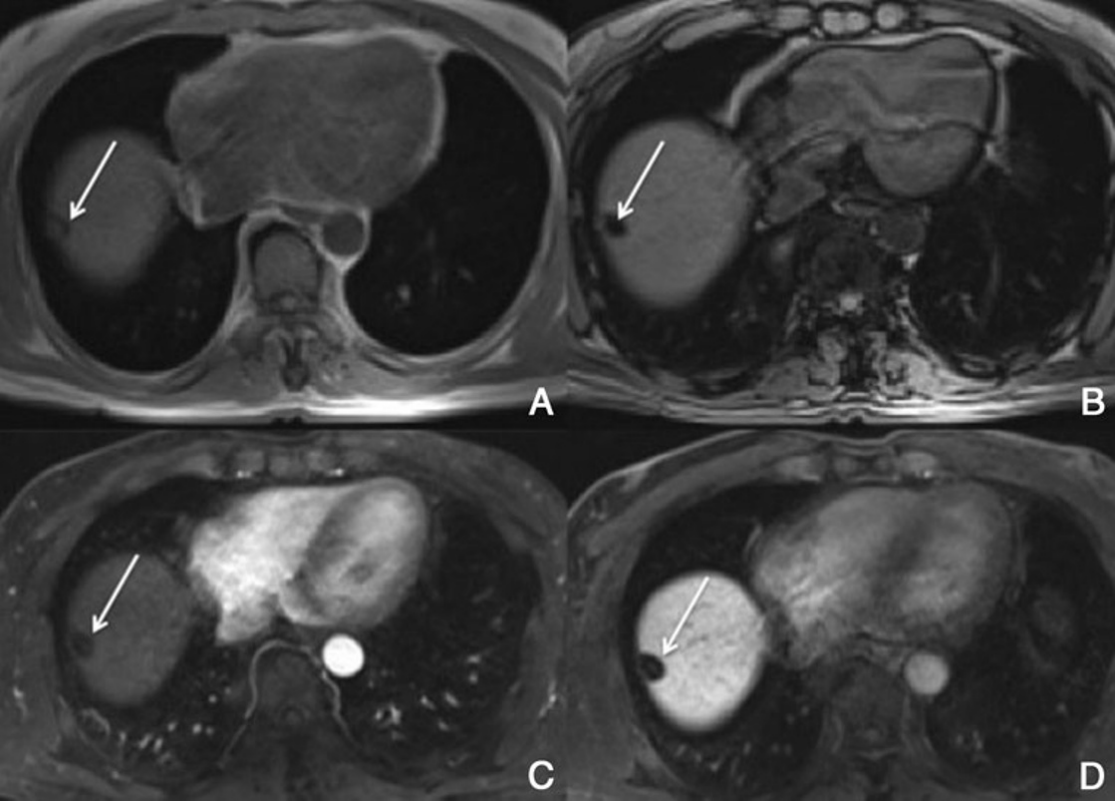
Pseudolipoma (Glisson’s capsule lipoma). **a** IP **b** OP **c** arterial **d** HCP. Small, no enhancing peripheral lesion (*arrow*), losing internal signal in OP, indicating the presence of intracellular fat. Histologically proven, after surgery, to be a pseudolipoma

**Fig. 25:**
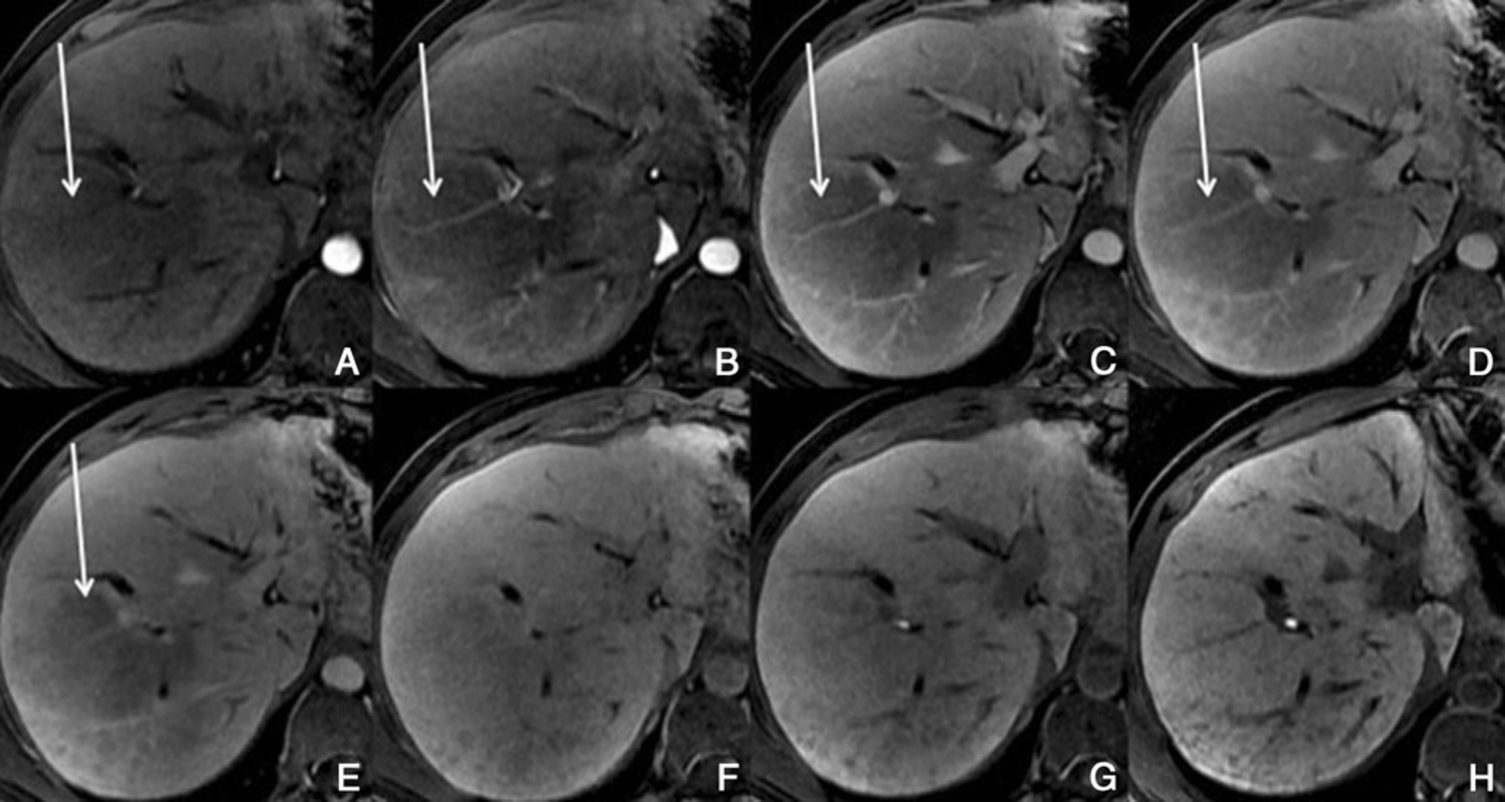
Inflammatory pseudotumour, in a 52-year-old man with PSC. **a, b** Arterial phase, **c** PVP, **d** 3 min, **e** 5 min, **f** HCP. Ill-defined hypointense area, in the right lobe (*arrows*), seen in the dynamic phases, not visualised in the HCP, proven to represent an inflammatory pseudolesion

**Fig. 26:**
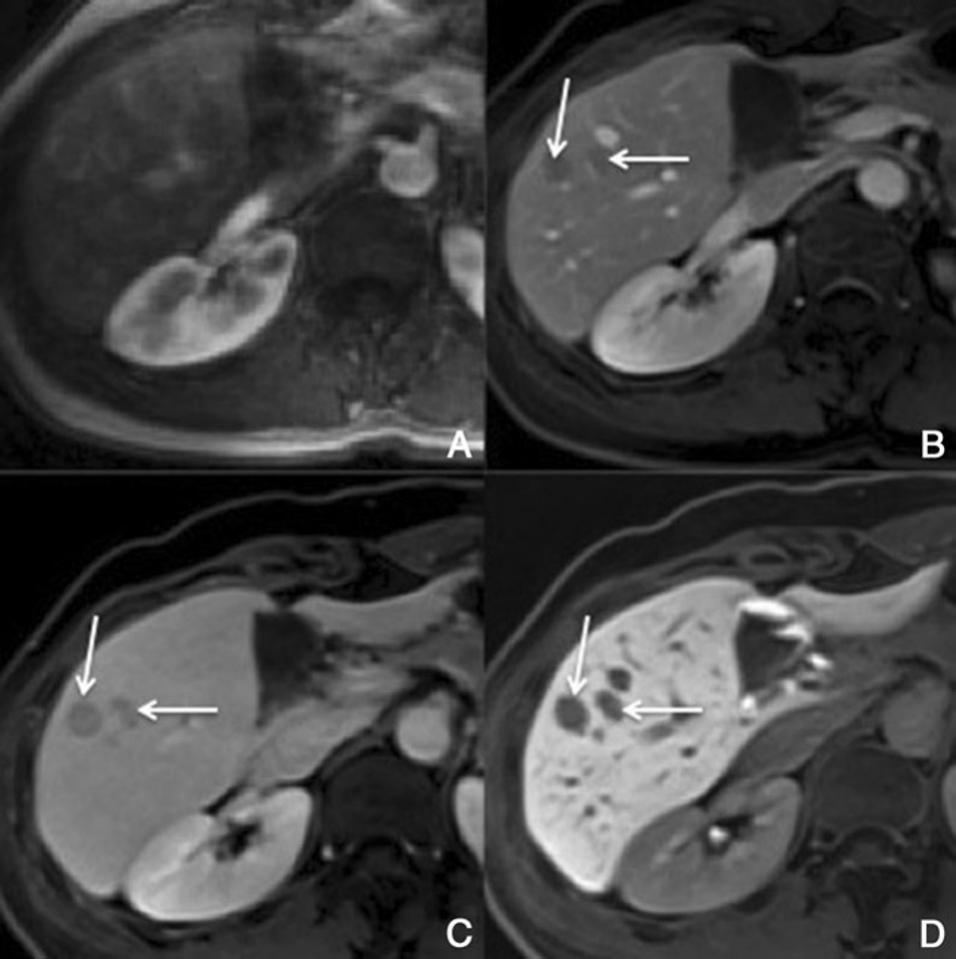
TP granulomas, in a 42-year-old man with active pulmonary TP. **a** Arterial phase, **b** PVP, **c** delayed phase, **d** HCP. There are two nodular lesions (*arrows*), with faint peripheral enhancement, that appear as defects in the HCP, as they have no hepatocytes to uptake Gd-EOB-DTPA

**Fig. 27:**
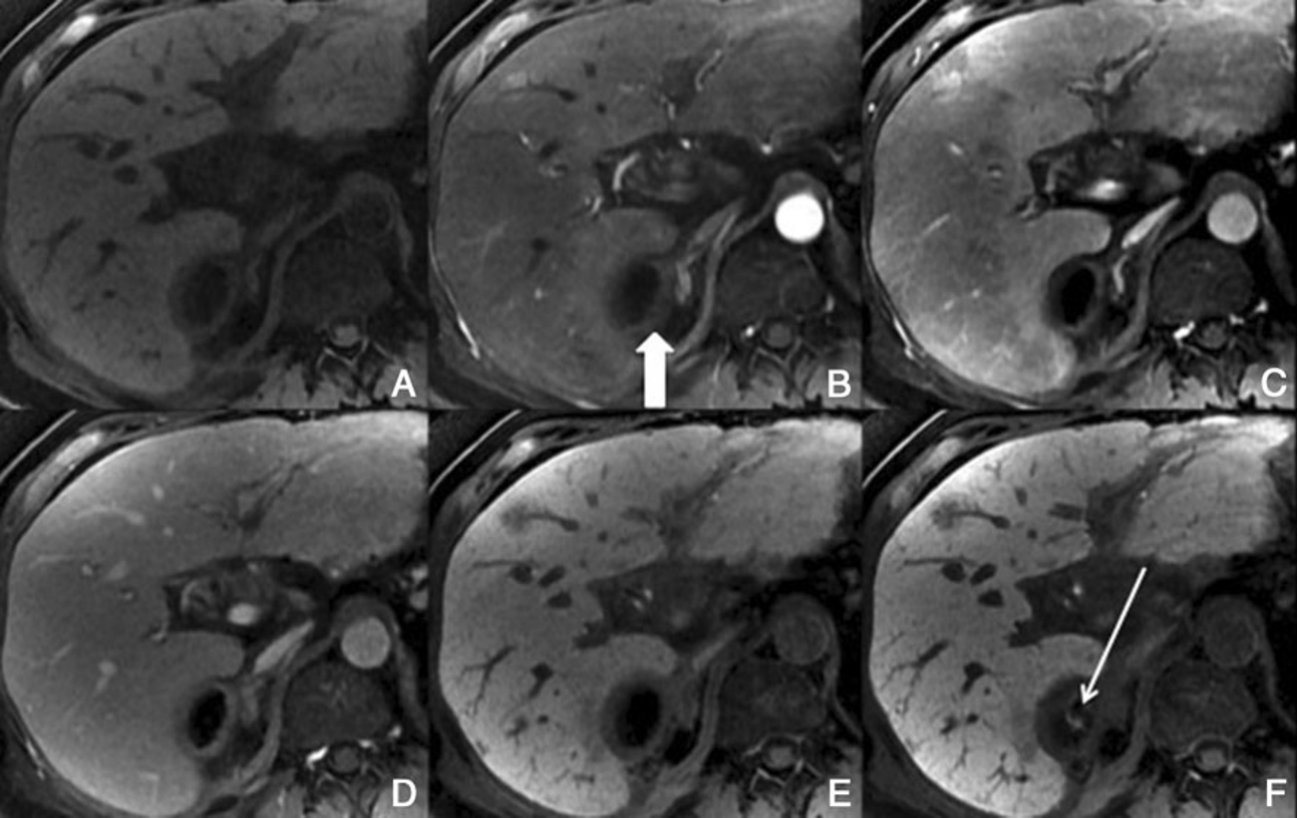
Liver abscess, in a 54-year-old man with history of choledocal litiasis. **a** Pre-contrast, **b, c** arterial, **d** 3 min, **e** 5 min, **f** HCP. On the posterior aspect of the liver, there is a thick-walled hypointense lesion (*solid arrow*), with no internal enhancement during the dynamic phases. Note the excretion of Gd-EOB-DTPA on the HCP, proving the connection of this abscess with the biliary tree

**Fig. 28:**
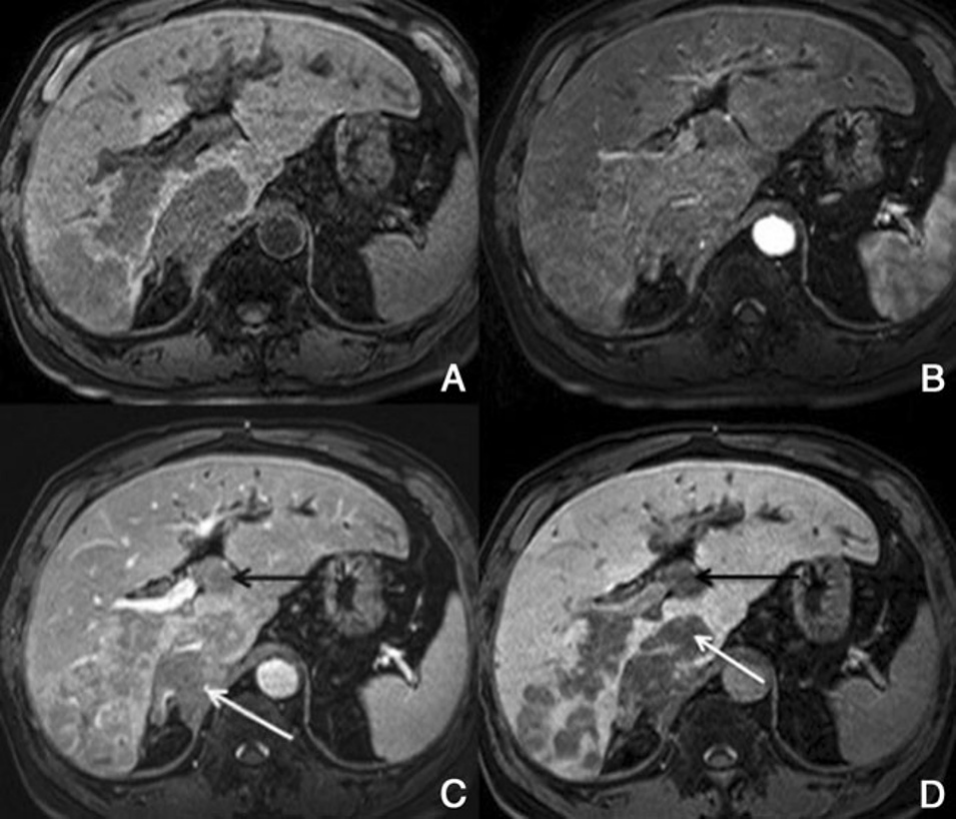
Liver Lymphoma, in a 39-year-old woman. **a** Pre-contrast, **b** arterial phase, **c** PVP, **d** HCP. Large infiltrative lesion in the right lobe (*arrows*), hypointense in the pre-contrast phase, with faint heterogeneous arterial enhancement, with washout in the PVP, not taking up Gd-EOB-DTPA in the HCP. Note involvement of the tumour along the periportal route (*black arrow*) and right adrenal gland (*white arrows*)

Focal hepatic lesions that show similar behaviour with ECF contrast agent and Gd-EOB-DTPA include hepatic cysts (Fig. 22), biliary hamartomas, biliary cystadenoma or carcinomas, angiomyolipoma (Fig. 23), lipoma (Fig. 24), inflammatory pseudotumour (Fig. 25), granulomatous disease (Fig. 26), hepatic abscesses (Fig. 27) and lymphoma (Fig. 28).

### Lesions in which the authors have no experience

The following lesions were never studied with Gd-EOB-DTPA-enhanced MRI: hematoma, peliosis, paranglioma and angiosarcoma.

### Summary

For a summary, see Tables 1 and 2.

**Table 1:** Patterns of enhancement and behaviour

Lesion	Arterial phase	PV phase	Delayed phase	Hepatobiliary phase
Benign	⇒	⇒	⇒	⇒
Benign	⇑	⇒	⇒	⇒
Benign	⇑	⇓	⇓	⇑
Indeterminate	⇓	⇓	⇓	⇓ or ⇑
Malignant	⇑	⇓	⇓	⇓
Malignant	⇓	⇑	⇓	⇓

**Table 2:** Specific lesions and imaging features

Lesion	T1-weighted imaging	T2-weighted imaging	Contrast dynamic imaging	Hepatobiliary phase	Pitfalls/comments
RN	Hypointense	Hypointense	Enhance	Isointense	
DN	Hyperintense	hypointense	Do not enhance	Hypo- or hyperintense	
HCC	Hypointense	Hyperintense	Usually intense enhancement with washout	Variable	Some HCCs will uptake Gd-EOB-DTPA in the hepatobiliary phase.
FL-HCC	Hypointense	Hyperintense with hypointense central scar	Enhances, hypovascular scar	Heterogeneous enhancement	
THID	No alteration	No alteration	Hypervascular lesion, fades in PV phase	No alteration	
FNH	Hypo- to isointense	Hyper- to isointense. Central scar hyperintense	Arterial uniform enhancement. Scar enhances in delayed imaging	Iso- to hyperintense	Gd-EOB-DTPA permits the evaluation of the internal architecture (with the internal reticulation), which permit more confidence in the diagnosis.
Adenoma	Hyper- to isointense	Moderately hyperintense	Enhance in arterial phase	Hypointense	
Focal hypereosinophilic necrosis	Hypointense	Hyperintense	Enhancement in PV phase	Mixed intensity	Difficult to rule out metastases
Confluent fibrosis	Hypointense	Hyperintense	Delayed enhancement	Hypointense	
Haemangioma	Hypointense	Markedly hyperintense	Progressive centripetal enhancement	Hypointense	Pseudowashout
Focal fat/focal sparing	Hyperintense loosing signal in opposed phase imaging/ hypointense	Hypointense			
Cholangiocarcinoma	Hypointense	Hyperintense	Delayed enhancement	Hypointense	
Hypervascular					
metastases	Hypointense	Iso- to hyperintense	Arterial enhancement. Peripheral washout	Hypointense	Central pooling of Gd-EOB-DTPA in the hepatobiliary phase
Hypovascular metastases	Hypointense	Iso- to hyperintense	No significant enhancement	Hypointense	
Foreign body reaction	Hypointense	Hyperintense with hypointense bands	Peripheral enhancement	Hypointense	
Cyst	Hypointense	Hyperintense	No enhancement	No enhancement	
Hamartoma	Hypointense	Hyperintense	No enhancement	No enhancement	
Cystadenoma/cystadenocarcinoma	Hypointense	Hyperintense	Nodular parietal enhancement points to carcinoma	No enhancement	
Angiomyolipoma	Containing macro or microscopic fat suppressed with fat suppressing or opposed phase imaging		Intense arterial enhancement		
Lipoma	Hyperintense, with loss of signal with fat suppression	Hyperintense	No enhancement	No enhancement	
Inflammatory pseudotumour	Hypointense	Hyperintense	Peripheral or delayed enhancement	No enhancement	
Granuloma	Hypointense	Hypointense	May show delayed enhancement	No enhancement	
Abscess	Hypointense	Hyperintense	Presence of a enhancing capsule	No enhancement	
Lymphoma	Hypointense	Variable	Faint peripheral enhancement	No enhancement	
